# Construction and comprehensive analysis of a novel prognostic signature associated with pyroptosis molecular subtypes in patients with pancreatic adenocarcinoma

**DOI:** 10.3389/fimmu.2023.1111494

**Published:** 2023-02-03

**Authors:** Qian Huang, Xingyu Peng, Qingqing Li, Jinfeng Zhu, Ju Xue, Hua Jiang

**Affiliations:** ^1^ Department of General Practice, Shanghai East Hospital, Tongji University School of Medicine, Shanghai, China; ^2^ Department of Geriatrics, Shanghai East Hospital, Tongji University School of Medicine, Shanghai, China; ^3^ Department of General Surgery, The Second Affiliated Hospital of Nanchang University, Nanchang, China; ^4^ Department of Pathology, Hunan Provincial People’s Hospital, The First Affiliated Hospital of Hunan Normal University, Changsha, China

**Keywords:** pancreatic cancer, pyroptosis molecular subtypes, prognostic signature, tumor immune microenvironment, tumor stemness, chemotherapeutic drug sensitivity, small molecule compounds

## Abstract

**Background:**

Treatment of cancer with pyroptosis is an emerging strategy. Molecular subtypes based on pyroptosis-related genes(PRGs) seem to be considered more conducive to individualized therapy. It is meaningful to construct a pyroptosis molecular subtypes-related prognostic signature (PMSRPS) to predict the overall survival (OS) of patients with pancreatic adenocarcinoma(PAAD) and guide treatment.

**Methods:**

Based on the transcriptome data of 23 PRGs, consensus clustering was applied to divide the TCGA and GSE102238 combined cohort into three PRGclusters. Prognosis-related differentially expressed genes(DEGs) among PRGclusters were subjected to LASSO Cox regression analysis to determine a PMSRPS. External cohort and *in vitro* experiments were conducted to verify this PMSRPS. The CIBERSORT algorithm, the ESTIMATE algorithm and the Immunophenoscore (IPS) were used to analyze the infiltrating abundance of immune cells, the tumor microenvironment (TME), and the response to immunotherapy, respectively. Wilcoxon analysis was used to compare tumor mutational burden (TMB) and RNA stemness scores (RNAss) between groups. RT-qPCR and *in vitro* functional experiments were used for evaluating the expression and function of SFTA2.

**Results:**

Based on three PRGclusters, 828 DEGs were obtained and a PMSRPS was subsequently constructed. In internal and external validation, patients in the high-risk group had significantly lower OS than those in the low-risk group and PMSRPS was confirmed to be an independent prognostic risk factor for patients with PAAD with good predictive performance. Immune cell infiltration abundance and TME scores indicate patients in the high-risk group have typical immunosuppressive microenvironment characteristics. Analysis of IPS suggests patients in the high-risk group responded better to novel immune checkpoint inhibitors (ICIs) than PD1/CTLA4. The high-risk group had higher TMB and RNAss. In addition, 10 potential small-molecule compounds were screened out. Finally, we found that the mRNA expression of SFTA2 gene with the highest risk coefficient in PMSRPS was significantly higher in PAAD than in paracancerous tissues, and knockdown of it significantly delayed the progression of PAAD.

**Conclusions:**

PMSRPS can well predict the prognosis, TME and immunotherapy response of patients with PAAD, identify potential drugs, and provide treatment guidance based on individual needs.

## 1 Introduction

Pancreatic adenocarcinoma (PAAD) is one of the most lethal malignancies, with a 5-year survival rate of only 11% after diagnosis, and is expected to become the second leading cause of cancer death in the United States over the next few decades (1, 2). PAAD is difficult to detect in the early stage, and metastatic spread often occurs when it is detected. It is estimated that only about 15-20% of patients with PAAD currently have the chance to have a radical cure through surgery, and the majority of patients’ prognoses are improved by adjuvant systemic chemotherapy (1). Despite this, patients with PAAD who receive a combination of surgery, chemotherapy, and radiotherapy benefit only in a small percentage of cases (3). In addition, four major immune defects also prevent the majority of patients with PAAD from responding optimally to emerging immunotherapies, and they are the absence of effective intratumoral T cells, heterogeneous dense stroma, immunosuppressive tumor microenvironment, and a lack of tumor-killing T cells (4). In short, the poor efficacy and prognosis of patients with PAAD make it particularly important to construct a new prognostic signature and guide individual treatment plans.

Pyroptosis is a new form of programmed cell death, which manifests as swelling of cells until their membrane ruptures, activating a strong inflammatory response by releasing cellular contents. It is biochemically characterized by inflammasome formation, activation of the caspase family and gasdermin, formation of membrane pores, and release of numerous proinflammatory cytokines, such as IL-1β and IL-18 (5–8). Pyroptosis-induced inflammatory responses can protect the host from microbial infection through classical or non-classical pathways (9). In addition to infectious diseases, more and more studies have recently confirmed the role of pyroptosis in malignant tumors’ s occurrence and development (10, 11). Chen et al. reported that the combination of ruthenium (II) polypyridyl complex Δ-Ru1 and Taxol enhance the anti-cancer effect on Taxol-resistant cancer cells through Caspase-1/GSDMD-mediated pyroptosis (12). Zhang et al. showed that injecting intratumorally with DM-αKG significantly inhibited tumor growth and metastasis through caspase-8-mediated GSDMC-dependent pyroptosis (13). Moreover, commonly used chemotherapeutic drugs, such as cisplatin and paclitaxel, are also effective at inhibiting tumor proliferation and metastasis by inducing pyroptosis (14, 15).

In recent years, gene expression signatures based on pyroptosis-related genes(PRGs) have been reported to predict the prognosis of many cancers, including bladder cancer (16), hepatocellular carcinoma (17), uveal melanoma (18), PAAD (19) among others. However, identification of molecular subtypes on the basis of gene expression seems to be a promising new approach as it helps to rapidly identify cancer features and derive the most appropriate treatment strategies (20). A high quality review also confirmed that the classification of pancreatic tumors based on their molecular characteristics is important to improve the accuracy of clinical treatment decisions (21).

The intervention of pyroptosis identifies a new area of research for the prognosis and treatment of patients with PAAD. Therefore, we established a novel prognostic signature based on the pyroptosis molecular subtype to predict OS and better guide treatment in patients with PAAD.

## 2 Materials and methods

### 2.1 Collection of data

The RNA expression data and clinical data of four independent PAAD cohorts(TCGA-PAAD, n = 182 [178 tumors, 4 normal]; GSE102238, n = 100 [50 tumors, 50 normal]; GSE57495, n=63[63 tumors]; ICGC-PACA-CA, n = 262 [262 tumors]) were downloaded from the following public databases: The Cancer Genome Atlas (TCGA) (https://portal.gdc.cancer.gov/), the Gene Expression Omnibus (GEO) (https://www.ncbi.nlm.nih.gov/geo/) and the International Cancer Genomics Consortium (ICGC) (https://dcc.icgc.org/). Cell lines gene expression matrices for primary PAAD were obtained from the Cancer Cell Line Encyclopedia(CCLE) dataset (https://portals.broadinstitute.org/ccle/about). TCGA and GSE102238 cohorts are used to build the signature, and the ICGC and GSE57495 cohorts are used to externally verify the signature. To merge TCGA with the GSE102238 cohort, we proceeded as follows. First, we converted the fragments per kilobase of transcript per million value of the RNA expression data of the TCGA cohort to transcripts per kilobase million value. Then, the intersection genes of TCGA and GSE102238 cohorts were extracted, and the corresponding expression data were matched. Next, we removed normal samples of the TCGA and GSE102238 cohorts, converted the TCGA TPM data to log_2_(TPM+1) data, and normalized the data from the GSE102238 cohort. Finally, we merged the processed TCGA with the GSE102238 cohort into a cohort, and used the ComBat function of the “SVA” package to remove batch effects on the merged data(n=227) (22). To reduce the probability of non-cancer death, we excluded patients with PAAD with a survival time of<30 days.

### 2.2 Establishment of molecular subtypes based on PRGs expression and screening of differentially expressed genes

52 PRGs (**Supplementary Table 1**) were from previous researches (23–25). The TCGA and GSE102238 cohorts were combined into one cohort (n=227), and the expression data of 23 PRGs were obtained after matching with 52 PRGs. k-means clustering algorithm was used to obtain the different molecular subtypes associated with PRGs expression. Every molecular subtype was named pyroptosis-related gene cluster (PRGcluster). Single sample gene set enrichment analysis algorithm (ssGSEA) and Normalized enrichment score (NES) were used to quantify the infiltrating abundance of 23 immune cells in different PRGclusters (26). Principal component analysis (PCA) can determine whether the three PRGclusters can be separated. Gene set variation analysis (GSVA) enrichment analysis was used to discover the underlying biological functions between PRGclusters. |log_2_FC|>0.585 and false discovery rate (FDR)<0.05 were considered as the criteria for screening differentially expressed genes (DEGs) among the three PRGclusters (27).

### 2.3 Gene enrichment analysis

The Gene Ontology(GO) and Kyoto Encyclopedia of Genes and Genomes(KEGG) gene enrichment analysis was used to explore the relevant cytological functions and pathways of DEGs. q-value<0.05 was the cutoff criterion for determining whether a gene is significantly enriched.

### 2.4 Establishment and identification of prognostic signature

Univariate Cox regression analysis helped screen prognosis-related differentially expressed genes (PRDEGs). The TCGA+GSE102238 cohort composed of transcriptome data of PRDEGs and the corresponding survival information were randomly divided into the training cohort and testing cohort. The least absolute shrinkage and selection operator (LASSO) Cox regression analysis was performed on PRDEGs in the training cohort to construct a refined prognostic signature (28). Here is the formula for the risk score:


$$\text{Risk score}=\sum_{i=1}^{n}coef\left(\text{Xi}\right)\times exp\left(\text{Xi}\right)$$

“Coef(Xi)”, “exp(Xi)”, and “n” represent the gene coefficient, expression level, and number of genes, respectively. The median risk score of the training cohort divided patients in the training cohort and validation cohorts into the low- and high-risk groups. Kaplan-Meier survival curves and time-dependent ROC were used to evaluate the prognostic predictive performance of pyroptosis molecular subtypes-related prognostic signature (PMSRPS). To analyze PMSRPS’ independent prognostic performance, univariate and multivariate Cox regression analyses were performed.

### 2.5 Immune and mutant landscapes between different risk groups

The Cell-type Identification By Estimating Relative Subsets Of RNA Transcripts (CIBERSORT) algorithm was used to quantify immune cell infiltration. Based on the Estimation of STromal and Immune cells in Malignant Tumours using the Expression data (ESTIMATE) algorithm (29), we calculated the stromal score, immune score, ESTIMATE score (sum of Stromal and immune scores) for each sample to quantify tumors Microenvironment. Potential immune checkpoint molecules refer to published papers (30, 31) (n=40, **Supplementary Table 2**). Immunophenoscore (IPS) was downloaded from The Cancer Immune Atlas (TCIA) (https://www.tcia.at/home) to compare responses to immune checkpoint inhibitors(ICIs) across different risk groups. TMB, the number of gene mutations, and the type of gene mutation were derived from the somatic mutation data(n=182) of the TCGA database. The gene copy number (n=185) was downloaded from the specific website(https://xena.ucsc.edu) to observe the copy number variation of PRGs. The RNA stemness scores (RNAss) were downloaded from the Pan-Cancer Atlas Hub (https://pancanatlas.xenahubs.net) (32).

### 2.6 Chemotherapeutic drug sensitivity and Identification of small-molecule compounds

Half maximal inhibitory concentration (IC50) was using for predicting the sensitivity of chemotherapy drugs in the high- and low-risk groups. |log_2_FC|>0.585 and FDR<0.05 were the screening criteria for DEGs between high- and low-risk groups. After uploading the up-regulated genome (log_2_FC>0) and down-regulated genome (log_2_FC<0) to the L1000FWD website (https://maayanlab.cloud/L1000FWD), a table including drug name, similarity score, and q-value among others will be obtained. Furthermore, 2D and 3D images of small molecule compounds were obtained from the PubChem website (https://pubchem.ncbi.nlm.nih.gov/).

### 2.7 Cell culture and transfection

The human PAAD cell line CFPAC-1 was derived from Procell (CL-0059, Wuhan, China) and was cultured in a special medium (Procell, CM-0059) in a 37°C, 5% CO2 incubator. CFPAC-1 cells have been identified by short tandem repeat (STR) sequences. The SFTA2-specific siRNA and negative control used in this study were from GenePharma (Shanghai, China). The sequences of the siRNAs are shown in **Supplementary Table 3**. CFPAC-1 cells were transfected using Lipofectamine 3000 reagent (Invitrogen, Waltham, USA) according to standard guidelines.

### 2.8 Samples and real-time quantitative PCR

The tumor tissue and paired adjacent tissue of PAAD diagnosed after operation were collected and stored at -80°C. Ethical approval was obtained from the Medical Research Ethics Committee of the Second Affiliated Hospital of Nanchang University. Consistent with a previous study (33), total RNA was extracted from tissues and transfected cells, respectively, and reverse transcription and real-time PCR were performed. The following primers used in the experiments are also shown in **Supplementary Table 3**.

### 2.9 *In vitro* functional experiments

#### 2.9.1 Cell counting kit-8

Cells in each group were seeded into 96-well plates at 5 × 10^3^ cells per well. After the cells were attached, adding 10 μl of CCK-8 reagent (GlpBio, GK10001, USA) to each well at 0, 24, 48 and 72 hours, respectively. After culturing for two hours, the absorbance value of each well was detected using a microplate reader at a wavelength of 450 nm.

#### 2.9.2 5-ethynyl-2’-deoxyuridine assay

Cells in each group were seeded into 96-well plates at 1.5 × 10^4^ cells per well. The next day, EdU staining was performed using an EdU kit (RiboBio, C10310-2, China) according to the manufacturer’s instructions. The percentage of EdU positive cells was calculated by the following formula: EdU positive rate=number of EdU positive cells/number of DAPI positive cells×100%.

#### 2.9.3 Transwell migration assay

Cells in each group were seeded into transwell chambers (Corning, 3422, USA) at 5 × 10^4^ cells per well. After 24 h, cells were fixed with 4% formaldehyde solution for 20 min, and then stained with 0.4% crystal violet solution for 20 min. The cells inside the cell were wiped with a moist cotton swab and allowed to dry, and the migrated cells outside the transwell chambers were counted using an inverted microscope.

### 2.10 Statistical analysis

This study used R software version 4.1.1 to analyze data. Log-rank test was used for survival analysis. Spearman correlation was used to analyze the correlation of risk scores with immune cell infiltration abundance, TMB, and ssRNA. Wilcoxon rank-sum test and chi-square test were used to judge the significant difference(*P*<0.05) between two or more groups.

## 3 Results

### 3.1 Effects of 52 PRGs on genetic variation, expression level and prognosis of PAAD

**Figure 1:**
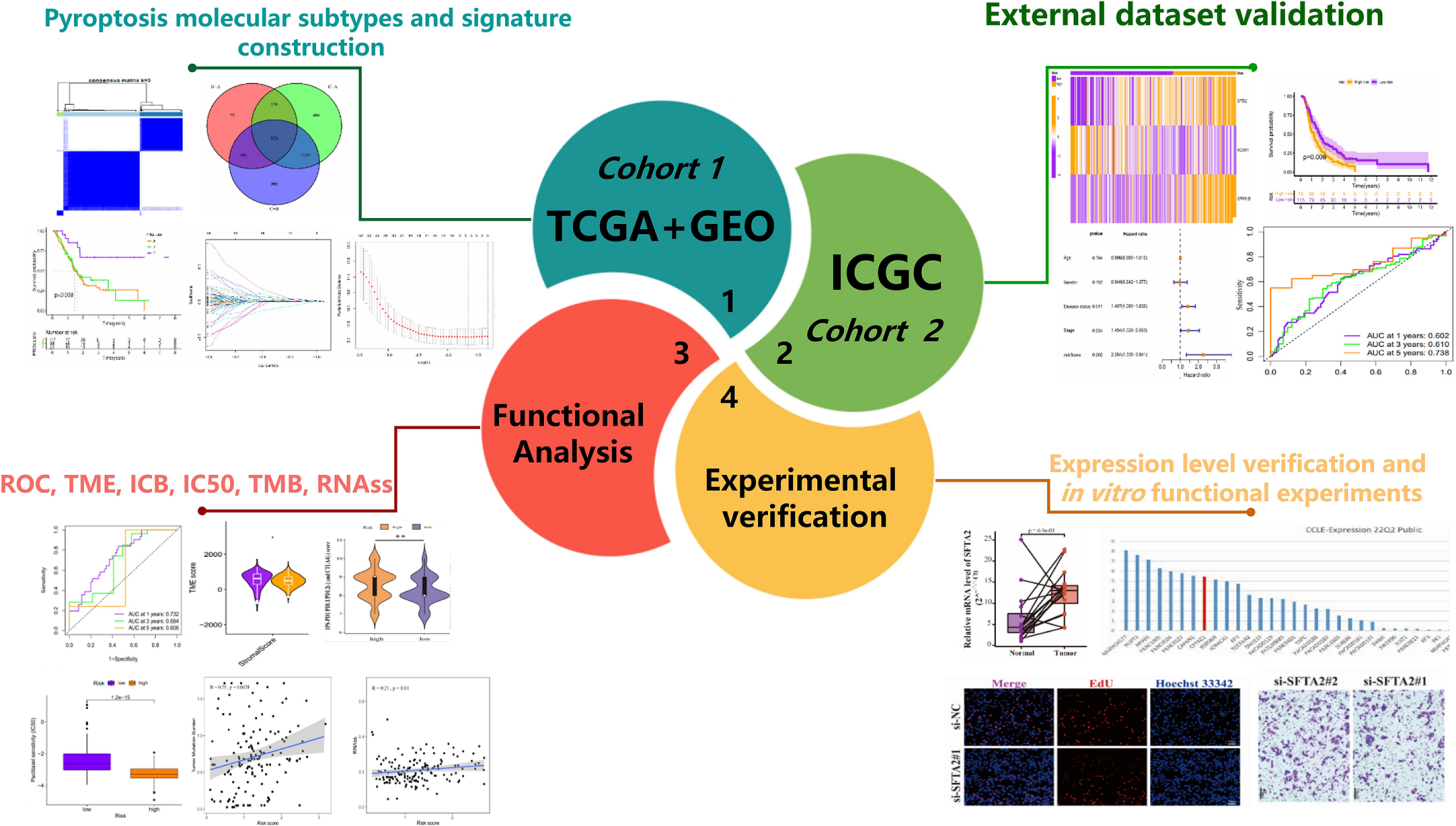
Rough flow chart of this study.

**Figure 2:**
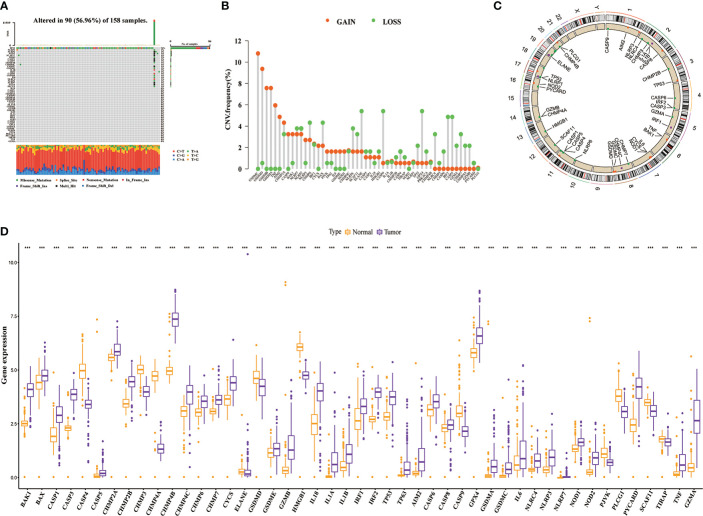
The genetic variation landscape of 52 pyroptosis-related genes (PRGs). **(A)** Somatic mutation frequency and type of PRGs. **(B)** Copy number variation frequency map of PRGs. **(C)** Copy number circle plot. **(D)** Differential expression of PRGs in the tumor and normal samples(Combined TCGA and GTEx databases). ***P< 0.001.

**Figure 1** shows the overall process of the study. Because of 52 PRGs obtained in this study were derived from ovarian cancer, esophageal adenocarcinoma and glioma studies (23–25), whether they have an effect on PAAD is uncertain. Therefore, we decided to conduct preliminary verification of these PRGs. Somatic mutation and gene expression data have been reported to help infer cancer progression (34). Beside gene mutation, about 95% of patients with PAAD have other genetic changes such as gene amplification and deletion (35). Therefore, we focused on analyzing the genetic variation and expression of PRGs in PAAD. The waterfall plot (**Figure 2A**) showed the frequency and type of somatic mutation of 52 PRGs in TCGA-PAAD. We found that among 158 samples, there are 90 mutations with a frequency of 56.96%, and TP53 demonstrated the highest mutation frequency, and most samples had the nonsense mutation(a type of point mutation in which a change in a base mutates a codon representing an amino acid into a stop codon, resulting in premature termination of peptide synthesis). The copy number variation (CNV) is an important basis and source of genetic variation (36). **Figure 2B** revealed that CNV changes occurred in all 52 PRGs. Of these, 27 PRGs(51.92%) were dominated by copy loss, 18 PRGs(34.62%) were dominated by copy gain, and the remaining PRGs had the same copy loss and copy gain frequency. The CNV of these genes on the chromosome is shown in **Figure 2C**. Next, we compared the expression of 52 PRGs in PAAD and normal pancreatic tissues by combining TCGA and the Genotype-Tissue Expression project (GTEx)(https://xenabrowser.net/datapages/) database. The results showed that 36 PRGs were significantly overexpressed and only 11 underexpressed in PAAD (**Figure 2D**). We also explored the prognostic value of PRGs matched with survival information and found that the expression of the majority of PRGs (16/23) correlated with the prognosis of patients with PAAD (**Supplementary Figure 1**). In total, the genetic variation, expression level and prognostic changes presented by 52 PRGs in PAAD indicated that they may play a role in the occurrence and development of PAAD, and it is convincing to use them for further analysis.

### 3.2 Identification of pyroptosis molecular subtypes and extraction of DEGs

**Figure 3:**
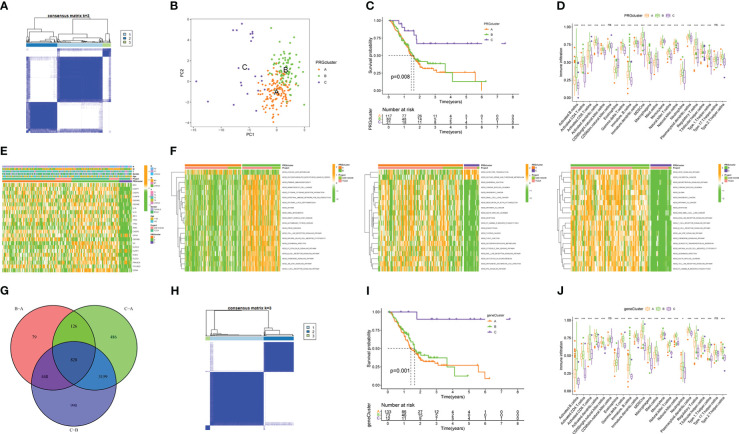
Molecular subtypes based on the expression of 23 PRGs and identification of DEGs. **(A)** 227 PAAD samples were divided into three PRGclusters using the Euclidean algorithm. **(B)** Principal component analysis (PCA) among three PRGclusters. **(C)** Kaplan-Meier survival analysis among three PRGclusters. **(D)** Comparison of immune cell infiltration abundance among three PRGclusters through ssGSEA analysis. **(E)** Heatmap showing clinicopathological information of three PRGclusters. **(F)** GSVA analyses of any two PRGclusters. **(G)** The Venn map of 828 differentially expressed genes (DEGs). **(H)** A sample of 276 DEGs associated with prognosis was divided into three geneClusters using the Euclidean algorithm. **(I)** Kaplan-Meier survival analysis among three geneClusters. **(J)** Differential expression of PRGs among three geneClusters. *P< 0.05, **P< 0.01, ***P< 0.001. ns, no statistical difference.

Prevalent intratumoral and intertumoral heterogeneity has been revealed an important cause of poor prognosis of PAAD. Therefore, in recent years, increasing evidence supports the subtyping of pancreatic tumors based on their molecular characteristics to improve the accuracy of clinical decision-making on treatment (21). Among them, transcriptome subtyping has been used by more and more people because of its unbiased classification that is robust and reproducible. Therefore, we conducted consensus clustering and typing of 23 PRGs that matched transcriptome data and found that the intergroup correlations were the lowest and the intragroup correlations were the highest when clustering variable k = 3, indicating that the joint cohort (n = 227, merged by TCGA and GSE102238) could be well clustered into three subtypes namely PRGclusters A, B, and C according to the expression of the 23 PRGs (**Figure 3A**). The results of PCA analysis also confirmed the rationality of this classification (**Figure 3B**). The results of the Kaplan-Meier survival analysis showed that the OS of PRGcluster C was significantly better than that of PRGclusters A and B (**Figure 3C**). The results of ssGSEA analysis showed that PRGcluster B had the highest infiltration abundance in most immune cells, which not only included anti-tumor immune cells, but also pro-tumor immunosuppressor cells and immune cells with function of anti-tumor and pro-tumor, followed by PRGcluster A and PRGcluster C (*P*<0.05, **Figure 3D**). Only the infiltrate abundance of Type.17.T.helper. cell and CD56dim.natural. killer(NK) did not differ significantly among the three PRGclusters. Next, we tried to find the correlation between the prognosis of different PRGclusters and immune cell infiltration and found that PRGcluster C had the least infiltration in some immunosuppressive cells that promoted tumors: MDSC, immature B cell, mast cell, plasmacytoid. dendritic cell, regulatory T cell(Tregs) and Type.2.T.helper.cell, which seem to help understand why PRGcluster C has the best prognostic effect. However, PRGcluster B has a lower prognosis than PRGcluster C despite having the highest invasion abundance among most antitumor immune cells. We speculate that this is the result of the checks and balances and interactions between different functional immune cells in PRGclsuter. Together these results suggest a complex association between immune cell infiltration and prognosis of different pyroptosis molecular subtypes of PAAD. The heatmap presents the clinicopathological features of the three PRGclusters (**Figure 3E**). In addition, we performed GSVA analysis on three PRGclusters and found that PRGcluster A was highly expressed in apoptosis, linoleic acid metabolism, and P53 signaling pathway; PRGcluster B was highly expressed in infection, immunity, pyroptosis, and various tumors; and PRGcluster C was up-regulated in olfactory transduction and glycine serine and threonine metabolism (**Figure 3F**). These results indicated that different types of pyroptosis had significantly different effects on the prognosis, immune activity, and biological function of patients with PAAD. To highlight these differences, a differential analysis of the expression levels of three PRGclusters was performed, and a total of 828 DEGs were obtained (**Figure 3G**).

### 3.3 The potential biological function of DEGs

**Figure 4:**
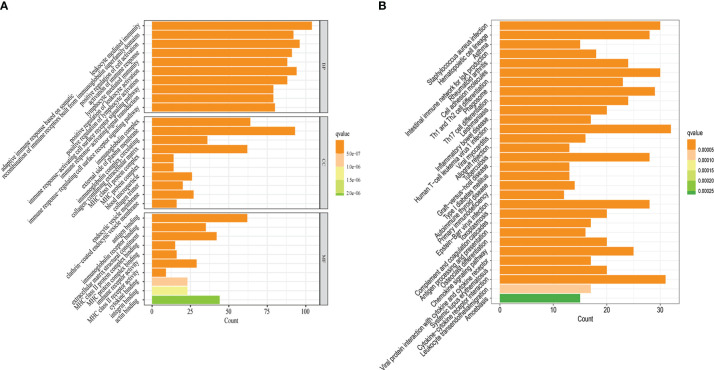
Potential biological functions of 828 DEGs. **(A)** The results of GO enrichment analysis. **(B)** The results of KEGG enrichment analysis. q-value<0.05 was the cutoff criterion for significant gene enrichment.

To better understand the potential function of 828 DEGs extracted, we performed GO and KEGG analyses. The GO enrichment results showed that DEGs were significantly enriched in leukocyte mediated immunity and lymphocyte mediated immunity (biological process), immunoglobulin complex, and external side of the plasma membrane (cellular component), as well as antigen binding and immunoglobulin receptor binding (molecular function) (**Figure 4A**). The KEGG enrichment results revealed that DEGs prominently enriched in staphylococcus aureus infection and hematopoietic cell lineage among others (**Figure 4B**). Therefore, DEGs may have biological functions that are relevant to cellular immunity and pyroptosis.

### 3.4 Verification of stability of pyroptosis molecular subtypes and establishment of PMSRPS

Next, we did a univariate Cox regression analysis for the DEGs combined with survival information and got 276 PRDEGs(**Supplementary Table 4**). Prior to grouping and modelling PRDEGs, it was realized that PMSRPS were established based on pyroptosis molecular subtypes(i.e. PRGclusters), failure to confirm the stability of subtypes inevitably affects the credibility of the signature, prompting us to perform another consensus clustering and typing of PRDEGs that match transcriptional data (37). The optimal cluster number supported the existence of three distinct and robust geneClusters in patients with PAAD (**Figure 3H**). Among these three geneClusters, the significant difference in OS was strikingly consistent with the result of PRGclusters (**Figure 3I**). Also, the infiltration abundance of 23 immune cells was highly in accordance with the differences among the PRGclusters (**Figure 3J**). More interestingly, we found that the majority of the geneClusters’ patients with PAAD came from the corresponding PRGclusters. In the survival analysis, the proportions of PRGclusters A, B and C in geneClusters A, B and C were 76.69%, 67.12% and 91.67%, respectively. In the immunoinfiltration analysis, the proportions of PRGclusters A, B and C in geneClusters A, B and C was 77.54%, 67.53% and 91.67%, respectively. This may provide a reasonable explanation for the high similarity in the survival and immune cell infiltration’ results between two clusters. All in all, these results suggest that pyroptosis molecular subtypes we identified are robust and reliable.

**Figure 5:**
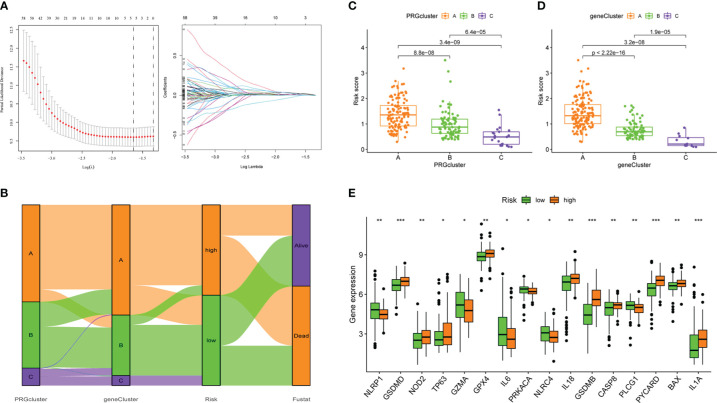
The establishment of PMSRPS and its relationship to molecular subtypes and PRGs. **(A)** Lasso regression analysis. **(B)** The Venn plot shows the relationship between molecular subtypes and risk score as well as survival state. **(C)** Distribution of risk scores among three PRGclusters. **(D)** Differences in risk scores among three geneClusters. **(E)** Differences in PRGs expression between the high- and low-risk groups. *P< 0.05, **P< 0.01, ***P< 0.001.

276 PRDEGs carrying transcriptomic data were subsequently matched with survival information to get a TCGA+GSE102238 cohort(n=218). The cohort was equally divided into a training cohort (n = 109) and a testing cohort (n = 109), and a significant difference was not found in proportion of clinical data and database sources between the two cohorts (**Table 1**). Next, we performed a LASSO Cox regression analysis on these PRDEGs in the training cohort (**Figure 5A**) and obtained a PMSRPS with the best fitting effect. The signature was composed of SFTA2, NCAM1, and SPRR1B. According to the risk coefficients of these three genes, we got a formula: Risk score=expression (SFTA2)×0.118+expression(NCAM1)×(-0.256)+expression(SPRR1B)×0.109. The median risk score of the training cohort, 1.053, divided the cohort of patients with PAAD into the high- and low-risk groups. The Venn diagram showed the relationship between clusters and risk scores, as well as survival status (**Figure 5B**). By comparison, we observed that both PRGcluster A and geneCluster A patients had significantly higher risk scores than other clusters (*P*<0.001, **Figures 5C, D**). In addition, the expression of 16 PRGs was also significantly different between the high- and low-risk groups (*P*<0.05, **Figure 5E**).

**Table 1:** Comparison of clinical data between the training and testing cohorts.

Clinical information		Training cohort (n=109)	Testing cohort (n=109)	*P*
Database	TCGA	85 (77.98)	86 (78.90)	1.00
	GSE102238	24 (22.02)	23 (21.10)	
Survival time, year	<=1	39 (35.78)	37 (33.94)	0.76
	>1 and<=5	67 (61.47)	67 (61.47)	
	>5	3 (2.75)	5 (4.59)	
Living state	ALIVE	43 (39.45)	55 (50.46)	0.13
	DEAD	66 (60.55)	54 (49.54)	
Age	<=65	59 (54.13)	58 (53.21)	1.00
	>65	50 (45.87)	51 (46.79)	
Gender	Female	46 (42.20)	51 (46.79)	0.59
	Male	63 (57.80)	58 (53.21)	
T stage	T1-2	20 (18.35)	17 (15.60)	0.72
	T3-4	88 (80.73)	91 (83.49)	
	Unknow	1 (0.92)	1 (0.92)	
M stage	M0	60 (55.05)	61 (55.96)	1.00
	M1	3 (2.75)	4 (3.67)	
	Unknow	46 (42.20)	44 (40.37)	
N stage	N0	37 (33.94)	38 (34.86)	0.96
	N1	70 (64.22)	68 (62.39)	
	Unknow	2 (1.83)	3 (2.75)	

P>0.05 means no significant statistical difference.

### 3.5 Internal validation of PMSRPS

**Figure 6:**
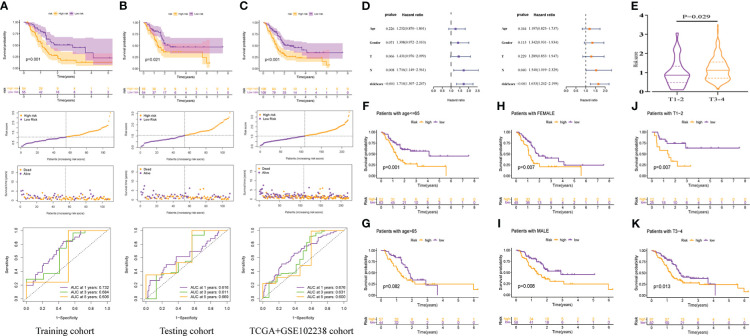
Internal cohorts validation of the predicted performance of PMSRP. **(A–C) **Comparison of overall survival (OS) between the high- and low-risk groups, and time-dependent ROC curves of risk scores in the training **(A)**, the testing **(B)**, and the TCGA+GSE102238 cohorts **(C)**. **(D)** Univariate and multivariate Cox regression analyses for independent prognostic performance assessment of PMSRPS. **(E)** Relationship between risk score and T stage. **(F–K)**The differences in OS between the high- and low-risk groups when patients with age<=65 **(F)** or >65 years **(G)**, the gender of female **(H)** or male **(I)**, and the tumor T stage of T1-2 **(J)** or T3-4 **(K)**.

The Kaplan-Meier survival curve showed that in the training cohort, the OS of patients with PAAD in the high-risk group was worse than that in the lower-risk group (*P*=0.001, **Figure 6A**). Also, the survival rate was obviously decreased in patients with PAAD with increasing risk scores (**Figure 6A**). Time-dependent ROC curves showed that the area under the curves (AUCs) for predicting 1-, 3-, and 5-year survival were 0.732, 0.684, and 0.606, respectively, in the training cohort (**Figure 6A**). As expected, we observed highly consistent results in the testing and TCGA+GSE102238 cohorts (all *P*<0.05, **Figures 6B, C**). This indicated that PMSRPS had a good predictive performance for the prognosis of patients with PAAD.

Subsequently, we verified the independence, clinical correlation and applicability of PMSRPS in the TCGA+GSE102238 cohorts. The results showed that risk scores could independently predict poor outcomes in patients with PAAD (*P*< 0.001, **Figure 6D**). That is, for all patients with PAAD, the higher the risk score, the worse the prognosis. There was also a significant relationship between risk scores and T stage, and we found that the risk scores of T3-4 were significantly higher than those of T1-2(*P*=0.029, **Figure 6E**), which also seemed to help explain the propensity of high risk scores for poor prognosis. In addition, results of applicability analysis showed that the survival rate was lower in the high-risk group than in the low-risk group when patients with age<=65 years (*P*= 0.001, **Figure 6F**) or >65 years(*P*= 0.082, **Figure 6G**), the gender of female or male (all *P*< 0.01, **Figures 6H, I**), and the tumor T stage of T1-2 or T3-4 (all *P*< 0.05, **Figures 6J, K**). This showed that PMSRPS could accurately predict and distinguish the prognosis of high- and low-risk groups at different ages, different genders, and different T stages. Taken together, the above results suggested that PMSRPS was an independent predictor of poor prognosis in patients with PAAD, and has general applicability.

### 3.6 External cohort verification and performance comparison of PMSRPS

**Figure 7:**
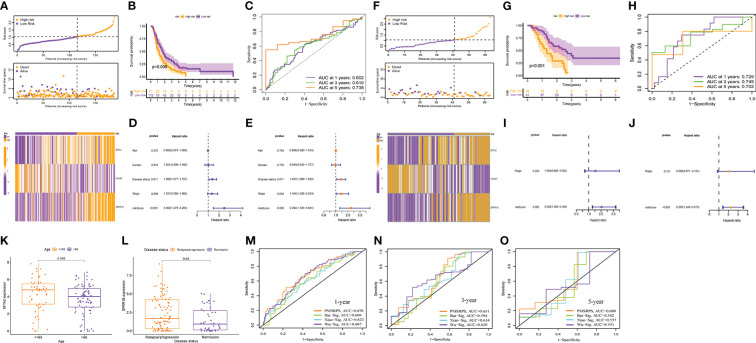
External cohort validation of the predicted performance of PMSRPS and performance comparison. **(A, F)** Risk grouping, survival status, and risk heatmap between the high- and low-risk groups in the ICGC **(A)** and GSE57495 **(F)** cohorts. **(B, G)** Comparison of OS between the high- and low-risk groups in the ICGC **(B)** and GSE57495 **(G)** cohorts. **(C, H)** Time-dependent ROC curves of PMSRPS in the ICGC **(C)** and GSE57495 **(H)** cohorts. **(D, E, I, J)** Results of independent prognostic analysis of PMSRPS in the ICGC **(D, E)** and GSE57495 **(I, J)** cohorts. **(K, L)** Results of external clinical correlation analysis for the high- and low-risk groups in the ICGC cohort. **(M–O)** Comparison of the performance of PMSRPS with Bai-Sig, Xiao-Sig and Wu-Sig in predicting 1- **(M)**, 3- **(N)**, 5-year **(O)** OS in patients with PAAD.

To confirm the robustness of PMSRPS, we calculated the risk scores of patients with PAAD in the ICGC and GSE57495 cohorts using the same formula, and divided patients into the high- and low-risk groups based on median risk score(1.053) (**Figures 7A, F**). Likewise, we observed that the number of survivors decreased with increasing risk scores (**Figures 7A, F**), and patients in the high-risk group had significantly lower OS than those in the low-risk group (all *P*< 0.01, **Figures 7B, G**). Time-dependent ROC curves showed that AUCs for PMSRPS to predict 1, 3 and 5-year survival rates for patients in ICGC cohort were 0.602, 0.610 and 0.738, respectively, while AUCs for predicting 1, 3 and 5-year survival rates for patients in GSE57495 cohort were all >0.7 (**Figures 7C, H**). Cox regression analysis revealed that risk score were independent prognostic factors in patients with PAAD (all *P*< 0.01, **Figures 7D, E, I**, **J**). Also, we verified the clinical relevance of PMSRPS in the ICGC cohort and found that the expression of the risk gene SFTA2 was higher in patients with PAAD aged 65 years and younger than in patients older than 65 years (**Figure 7K**). Moreover, the risk gene SPRR1B was highly expressed in the disease state of tumor recurrence/progression (**Figure 7L**).

Furthermore, to highlight the advantages of PMSRPS, we compared the performance of PMSRPS with three PAAD prognostic signatures recently published. The first is a pyroptosis-related risk signature constructed by Bai et al. (38). The second is a DNA-methylation-driven genes based prognostic signature (GPRC5A, SOWAHC, S100A14, ARNTL2) created by Xiao et al(39). The third is the m6A-related RNA signature(AP005233.2, AC092171.3, AC010175.1, CASC8, TP53TG1, SNAI3.AS1, FLRT1, AC022098.1, DCST1.AS1) constructed by Wu et al (40). The results showed that the AUCs of PMSRPS (0.676, 0.631, 0.600) for predicting the 1-, 3-, and 5-year overall survival of patients with PAAD were higher than those of Bai-Sig (0.609, 0.594, 0.542), Xiao-Sig (0.623, 0.614, 0.557) and Wu-Sig (0.667, 0.630, 0.551) (**Figures 7M–O**). This reveals that the predictive performance of PMSRPS is better than some existing prognostic signatures.

### 3.7 Prediction of tumor immune microenvironment and immunotherapeutic response in patients with PAAD by PMSRPS

**Figure 8:**
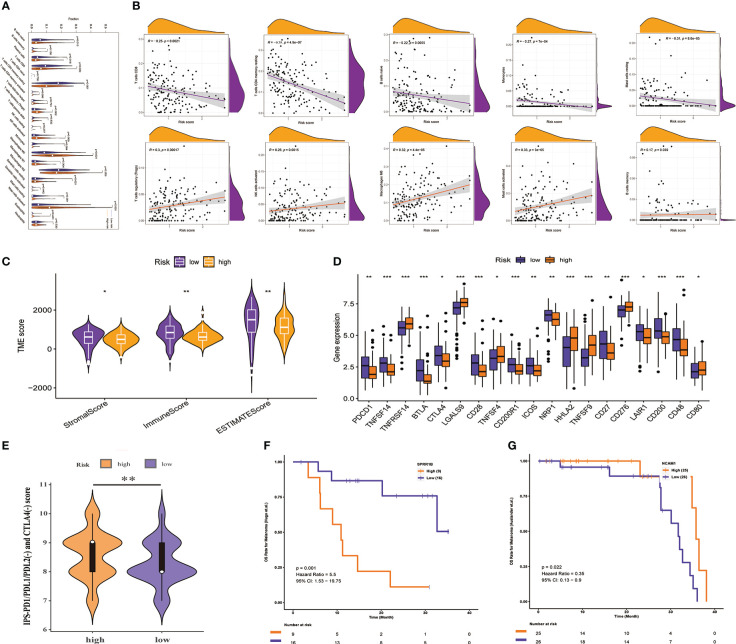
Prediction of TIME and immunotherapy response by PMSRPS. **(A)** Comparison of immune cell infiltration in the high- and low-risk groups. **(B)** Correlation of risk score with infiltrating abundance of 10 immune cells. **(C)** Comparison of tumor microenvironment scores in the high- and low-risk groups. **(D)** Differential expression of immune checkpoint molecules in the high- and low-risk groups. **(E)** Comparison of IPS-PD1/PDL1/PDL2(-) and CTLA4(-) scores between the high- and low-risk groups. **(F, G)** The predictive role of SPRR1B **(F)** and NCAM1 **(G)** genes in PMSRPS on existing immunotherapy cohorts was explored *via* a web server for Comprehensive Analysis on Multi-Omics of Immunotherapy in Pan-cancerimmune checkpoint inhibitors (CAMOIP). *P< 0.05, **P< 0.01, ***P< 0.001.

The TIME of dynamic evolution during tumor progression is an important histological feature of PAAD, which makes us eager to know whether there are TIME differences between high - and low-risk groups with different survival outcomes. We first analyzed the infiltration of immune cells. Results of the this study suggested that the abundance of Tregs, NK cells activated, Macrophages M0, and Mast cells activated infiltration was distinctly higher in the high-risk group than in the low-risk group (all *P*< 0.01, **Figure 8A**), and with increasing risk scores, their infiltration abundance increased prominently (all *P*< 0.01, **Figure 8B**). However, the abundance of T cells CD8, T cells CD4 memory resting, B cells naïve, Monocytes, and Mast cells resting infiltration in the high-risk group was significantly lower than that in the low-risk group (all *P*< 0.05, **Figure 8A**), and they were less abundant with greater risk scores (all *P*< 0.01, **Figure 8B**). In addition to infiltrating immune cells, infiltrating stromal cells are also vital components of tumor TIME, which together perturb the tumor signal and play an important role in cancer biology (29). Therefore, we used the ESTIMATE method to assess the distribution of these components between the high- and low-risk groups, and found that the stromal score, immune score, and estimated score were significantly lower in the high-risk group than in the low-risk group (all *P*<0.05, **Figure 8C**), which meant PMSRPS could accurately predict and distinguish TIME in the different risk groups, and that the high-risk group had higher tumor purity and more typical immunosuppressive “cold” tumor microenvironment. Furthermore, researchers found the expression of immune checkpoint molecules on a significant percentage of tumor cells, and they have been shown to promote epithelial-mesenchymal transformation, resistance to apoptosis and antitumor drugs, and propensity to spread and metastasize (41). In this study, we observed that about half (19/40) of the immune checkpoint molecules had significant expression differences between the two groups. Among them, a total of 12 genes, including PDCD1, BTLA, and CD28, were significantly highly expressed in the low-risk group, while 7 genes, including TNFRSF14, LGALS9, and CD276, were significantly highly expressed in the high-risk group (all *P*< 0.05, **Figure 8D**). To overcome the immunosuppressive power of checkpoint molecules, a new immunotherapy called ICIs works by blocking these immunosuppressive molecules and reactivating effector T cells to specifically kill tumor cells. Currently, the main ICIs used in clinical practice are CTLA4 inhibitors, PD-1 inhibitors and PD-L1 inhibitors. We evaluated the response of patients with PAAD in different risk groups to ICIs by IPS scores and found that the IPS (PD-1/PD-L1/PD-L2(-) and CTLA-4(-)) scores of patients in the high-risk group were significantly higher in the lower-risk group(*P*< 0.01, **Figure 8E**), rather than IPS-PD1/PDL1/PDL2 blocker score, IPS-CTLA4 blocker score, and IPS-CTLA4 and PD1/PDL1/PDL2 blocker score (**Supplementary Figure 2**). This suggested that patients with PAAD in the high-risk group might be more suitable for novel ICIs therapy rather than PD1/CTLA4 immunotherapy. Besides PMSRPS, we were surprised to find that the model gene had good predictive performance for the existing immunotherapy cohort *via* a web server for Comprehensive Analysis on Multi-Omics of Immunotherapy in Pan-cancerimmune checkpoint inhibitors (CAMOIP) (http://www.camoip.net/). The results showed that the highly expressed prognostic risk gene SPRR1B had significantly worse OS (HR: 5.5, *P*=0.001, **Figure 8F**) in the melanoma immunotherapy cohort of Hugo. et al. (42). And the highly expressed prognostic protective gene NCAM1 had significantly better OS (HR: 0.35, *P*=0.022, **Figure 8G**) in the melanoma immunotherapy cohort of Auslander et al. (43). Taken together, the above results manifested that PMSRPS might be a reliable tool for predicting immune activity and immunotherapy response in patients with PAAD.

### 3.8 Prediction of PMSRPS on other measures related to immunotherapy response

**Figure 9:**
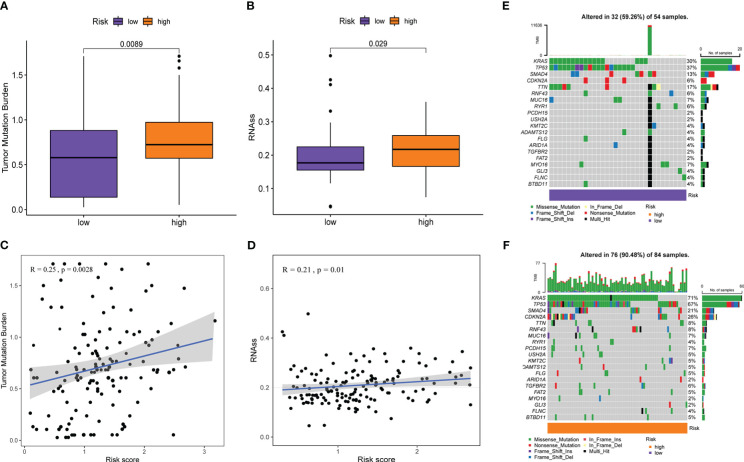
Prediction of PMSRPS on other measures related to immunotherapy response. **(A, B) **Comparison of tumor mutational burden(TMB) **(A)** and RNA stemness scores (RNAss) **(B)** between the high- and low-risk groups. **(C, D) **Correlation of risk score with TMB **(C)** and RNAss **(D)**. **(E, F) **Changes in gene mutation frequency and mutation type from the low-risk group **(E)** to the high-risk group **(F)**.

Although ICIs is considered a breakthrough in cancer treatment, the benefit to patients is limited, so it makes sense to understand the underlying indicators that influence response to ICIs treatment. Cancer stemness was reported to be significantly positively associated with ICIs resistance in cancer (44). TMB predicts clinical response to ICIs in a variety of tumors and is associated with improved survival after receiving ICIs (45). Genomic stability was the third proven predictor of ICIs response (46). Therefore, we explored these indicators in the high- and low-risk groups with different immunotherapy responses. As shown in **Figures 9A, B**, the TMB and RNAss of the high-risk group were visibly higher than those of the low-risk group (all *P*< 0.05), and TMB and RNAss had distinctly increased with the increase of the risk score (all *P ≤* 0.01, **Figures 9C, D**). In addition, the genomic mutation rate in the high-risk group was significantly higher than that in the low-risk group (90.48% [76/84] vs 59.26% [32/54], *P*<0.001, **Figures 9E, F**). Among them, gene mutation rates of the top two genes, KRAS and TP53, increased more significantly from the low- to the high-risk groups (KRAS: 71% [60/84] vs. 30% [16/54], *P*<0.001; TP53: 67% [56/84] vs 37% [20/54], *P*=0.001). These results suggest that high TMB, cancer stemness, and genomic instability are characteristic of high-risk patients compared with low-risk patients, which can help evaluate and predict immunotherapy effects in patients with different risk groups from multiple perspectives.

### 3.9 Sensitivity analysis of chemotherapeutic drugs and screening of small molecule drugs

**Figure 10:**
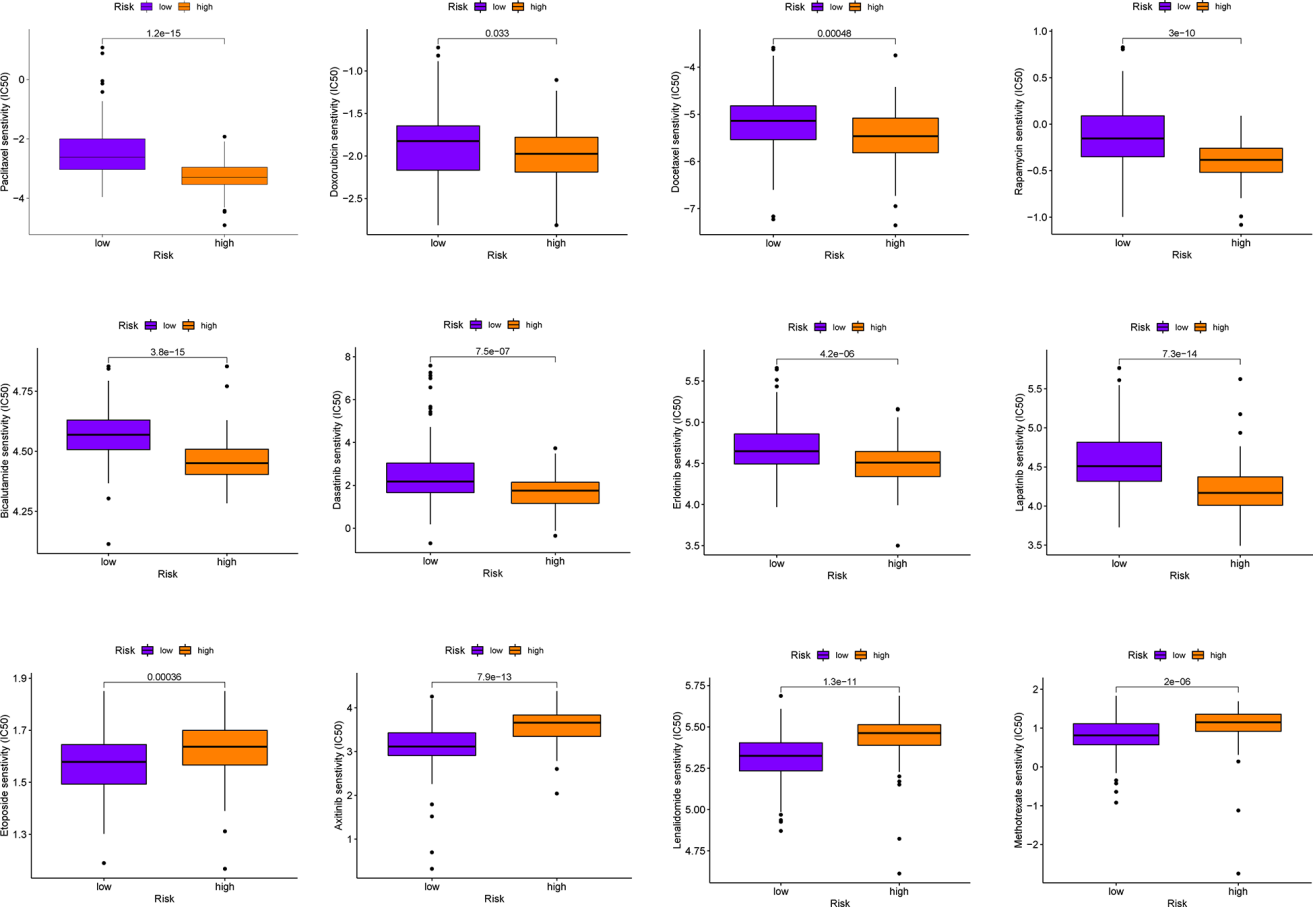
Comparison of sensitivities to commonly used chemotherapeutics for PAAD and other cancers in the high- and low-risk groups.

Immunotherapy has important therapeutic value for some malignancies, but in most phase I and II trials, It has failed to show good clinical efficacy in patients with PAAD when used alone, unless combined with chemotherapy (47). This suggests that it is necessary to simultaneously screen for more effective chemotherapy drugs. The results of chemotherapeutic drug sensitivity analysis in this study revealed that the high-risk group was more sensitive to commonly used chemotherapy drugs for PAAD (Paclitaxel, Doxorubicin, Docetaxel among others) than the low-risk group (all *P*< 0.05, **Figure 10**). Moreover, PMSRPS can predict chemotherapy responses in different risk populations to other cancers (Bicalutamide and Lenalidomide among others). (all *P*< 0.001, **Figure 10**).

In addition, to expand and search for potential small-molecule drugs for PAAD, we extracted DEGs between the high- and low-risk groups. Next, the up-regulated and down-regulated DEGs were uploaded separately to the L1000FWD website and matched with small molecule therapies. This study presented the 10 most important potential small molecule drugs, followed by similarity scores, q-values, and mechanism of action(**Table 2**). Among them, the top three drugs with negative similarity scores were tamoxifen, ZM-241385, and BRD-A24021119, which were expected to negatively regulate the expression of DEGs. 2D and 3D images of these three drugs could be seen in **Supplementary Figure 3**. These potential small-molecule drugs might reverse the high expression of high-risk group genes and guide the development of PAAD-targeted drugs. However, the exact efficacy of these drugs needs to be further confirmed in future prospective studies.

**Table 2:** Potential small molecule drugs screened for PAAD treatment.

Drug	Similarity score	q-value	Z-score	Combined score	MOA	Predicted MOA
tamoxifen	-0.5294	4.67E-08	1.59	-17.78	estrogen receptor antagonist, selective estrogen receptor modulator (SERM)	histamine receptor antagonist
ZM-241385	-0.5294	4.68E-08	1.66	-18.43	adenosine receptor antagonist	Aurora kinase inhibitor
BRD-A24021119	-0.5294	4.67E-08	1.77	-19.89	Unknown	PI3K inhibitor
BRD-K88622704	-0.5294	4.67E-08	1.73	-19.38	Unknown	MEK inhibitor
RU-24969	-0.4706	3.89E-07	1.69	-16.52	serotonin receptor agonist	histamine receptor antagonist
BRD-K62818989	-0.4706	3.89E-07	1.62	-15.66	Unknown	dopamine receptor antagonist
vemurafenib	-0.4706	3.89E-07	1.79	-17.27	RAF inhibitor	RAF inhibitor
gossypol	-0.4706	3.89E-07	1.84	-18.09	BCL inhibitor, MCL1 inhibitor	topoisomerase inhibitor
SB-525334	-0.4118	3.67E-06	1.67	-13.10	TGF beta receptor inhibitor	cyclooxygenase inhibitor
BRD-K90700939	-0.4118	3.86E-06	1.64	-12.79	Unknown	mTOR inhibitor

MOA, Mechanism of action.

### 3.10 Expression level and functional verification of SFTA2 gene

**Figure 11:**
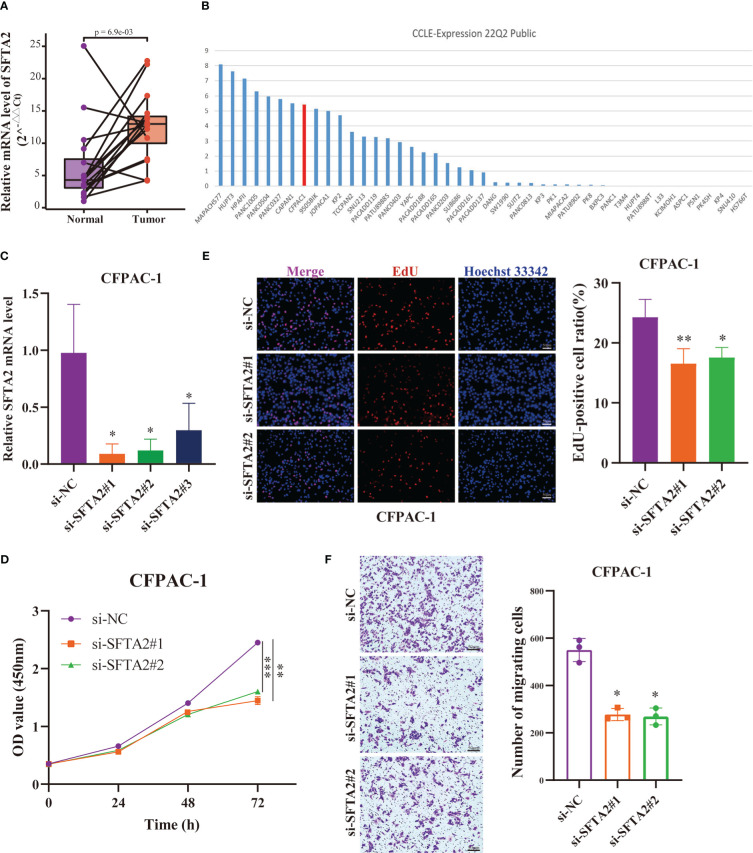
Expression and function of the highest-risk SFTA2 gene in PMSRPS. **(A)** qRT-PCR analysis of SFTA2 mRNA levels in randomly selected 16 pairs of PAAD tissues and corresponding adjacent tissues. **(B)** The Cancer Cell Lines Encyclopedia (CCLE) database was searched for PAAD CFPAC-1 cell lines suitable for SFTA2 intervention. **(C)** Validation of knockdown efficiency of SFTA2 interfering fragments in CFPAC-1 cells by qRT-PCR. **(D)** CCK-8 assay was used to assess the effect of SFTA2 silencing on CFPAC-1 cell viability. **(E)** EdU staining was used to assess the effect of SFTA2 silencing on the proliferative capacity of CFPAC-1 cells. **(F)** Transwell experiments were used to evaluate the effect of SFTA2 silencing on the migration ability of CFPAC-1 cells. *P< 0.05, **P< 0.01, ***P< 0.001.

Finally, to further verify the reliability of the PMSRPS, we performed expression and *in vitro* functional experiments on the SFTA2 gene with the highest correlation coefficient. As shown in **Figure 11A**, we found that SFTA2 was highly expressed in PAAD tissues compared with the corresponding paracancerous tissues (*P*<0.01). Further, we selected PAAD CFPAC-1 cell lines with high expression through CCLE database for SFTA2 gene interference (**Figure 11B**). **Figure 11C** shows that si-SFTA2#1 and si-SFTA2#2 had more obvious inhibitory effects, which was used for further functional experiments. Through CCK-8 experiments, we found that interfering with the expression of SFTA2 inhibited the cell viability of PAAD cells (**Figure 11D**). By EdU staining, we found that compared with the control group, the proliferation rate of the SFTA2-inhibited group was significantly reduced (**Figure 11E**). Through transwell migration experiments, we found that interfering with SFTA2 expression would inhibit the migration of PAAD cells (**Figure 11F**).

## 4 Discussion

The overall prognosis for patients with PAAD has long been disappointing (48). Authoritative cancer statistics in 2022 showed that PAAD has become the third leading cause of cancer-related death in both men and women (2). The poor prognosis of patients with PAAD is associated with the limited therapeutic response, and one possible reason for the poor therapeutic response is the ability of PAAD cells to avoid induction of death (49). Cell death is an important physiological process for maintaining tissue homeostasis, which can be divided into accidental cell death and regulated cell death (RCD) according to its occurrence rate and potential control (49, 50). RCD with clear mechanism of effect can be further subclassified into apoptotic and non-apoptotic subcategories (49). Since apoptosis is strongly resisted by cancer cells (51), targeting non-apoptotic cell death is considered a more promising therapeutic approach (49). Pyroptosis is a non-apoptotic death type characterized by the release of a large number of inflammatory factors, and its induced activation can produce powerful antitumor activity (52, 53). This is illustrated by the fact that wang et al. revealed that pyroptosis of less than 15% of tumour cells was sufficient to clear the entire 4T1 mammary tumour graftI by building a bioorthogonal chemical system (54). Moreover, Zhang et al’ s study showed that GSDME expression could not only enhance the phagocytosis of tumor-related macrophages, but also strengthen the number and function of NK cells and CD8+T cells infiltrated by tumors (55). In studies related to PAAD, Cui et al. demonstrated that MST1 inhibits pancreatic cancer progression *via* ROS-induced pyroptosis (56). Peng et al. showed that ICy OH produced by ICy Q could damage mitochondrial membranes, induce intracellular inflammatory responses, and selectively induce pancreatic cancer cell death *via* the cell pyroptosis pathway (a series of enzymatic reactions leading to the production of fragments of pyroptosis protein GSDMD-N) (57). Considering the promise of immunotherapy, researchers recently tested the immunotherapy effect of pyroptosis using the membrane targeted photosensitizer TBD-3C. The results showed that pyroptosis-aroused immunological responses could transfer the immunosuppressive “cold” tumor microenvironment(TME) into an immunogenic “hot” TME, which not only inhibited the growth of primary PAAD, but also attacked distant tumors (58). In brief, a new strategy to induce pyroptosis may provide more effective cancer treatment options (59). However, estimates of disease status relying solely on changes in gene expression are unstable (60). As a result, we sought to establish a prognostic signature through the analysis of pyroptosis molecular subtypes to aid in the accurate prediction of PAAD prognosis and guidance for individualized treatment.

We performed consensus clustering and typing of patients with PAAD based on the expression levels of 23 PRGs and obtained 828 DEGs. Through GO and KEGG enrichment analysis, we found that the potential biological functions of these DEGs were related to the occurrence of cellular immunity and pyroptosis. It has been reported that immune cells macrophages and neutrophils can interact to drive the pathogenesis of PAAD (61). Among them, M2 and a small fraction of M1 cells are not only unable to phagocytose tumor cells, but they also migrate to other tissues and organs without being killed(62). The immature c-Kit+ neutrophil subsets can generate ROS by participating in oxidative mitochondrial metabolism, thereby inhibiting the immune function of CD4^+^ T cells and promoting tumor progression (63). Furthermore, overactivation of the NLRP3 inflammasome may promote the development of hematopoietic malignancies (64).

Subsequently, we refined Lasso Cox regression analysis on the extracted 276 prognostic DEGs in the training cohort to obtain PMSRPS. Internal and external validation results showed that PMSRPS was an independent and effective prognostic tool for PAAD. Compared with some recently published PAAD prognostic signatures (38–40), PMSRPS had better predictive power in predicting 1-, 3-, and 5-year survival in patients with PAAD. PMSRPS consists of two risk prognostic genes (SFTA2 and SPRR1B) and one protective prognostic gene (NCAM1). SFTA2 is the gene with the highest risk factor in PMSRPS, and although it has been reported to be associated with poor prognosis of PDAC, experimental validation evidence is lacking (65). Therefore, this study verified for the first time that the expression of SFTA2 in PAAD tissues was higher than that in paired paracancer tissues in our own samples. It was further confirmed that the vitality, growth rate and migration ability of PAAD cells were significantly decreased after the SFTA2 expression level was knocked down. In addition, SPRR1B was a prognostic or diagnostic biomarker for various malignancies such as PAAD, lung adenocarcinoma, metastatic cutaneous melanoma, and oral squamous cell carcinoma among others (66–69). NCAM1 was confirmed to be up-regulated mediated by miR-141-3p and inhibited ameloblastoma cell migration (70).

Then, we used PMSRPS to predict the TIME of different risk groups and found that the high-risk group presented a typical tumor immunosuppressive microenvironment. This was reflected in less T cells CD8 and T cells CD4 memory resting as well as more Tregs and macrophage M0 in the high-risk group. Studies have shown that higher CD8 T cell infiltration in PDAC is strongly associated with long-term survival (71). In contrast, Tregs are important factors in maintaining immune tolerance, not only suppressing effector cells within the tumor but also restricting antitumor immune responses by interacting with stroma, vasculature, and lymphatic vessels (72). Also, M0 macrophages can differentiate into M2 macrophages in the presence of M-CSF, IL-4, or IL-10 and promote immune escape through the high expression of PD-L1, IL-10, or TGFβ (73). In addition, mast cells are key regulators of inflammation and immunosuppression (74). Besides suppressing anti-tumor immunity by releasing anti-inflammatory cytokines such as IL-10 and TGFβ, they can provide oxygen for tumor growth by regulating angiogenesis (75, 76). Excitingly, in this study, PMSRPS was confirmed to predict the expression of multiple immune checkpoint molecules in different risk groups and showed that the high-risk group had higher IPS (PD-1/PD-1/PD-1/PD-1/PD-1) and CTLA-4(-)) scores compared with the low-risk group. This suggests that PMSRPS may be a potential biomarker for predicting response to novel immunotherapies in PAAD.

Cancer stem cells with self-renewal ability can induce tumor metastasis and recurrence and are contribute to drug resistance (77, 78). Tumor stemness leading to immunotherapy resistance can be attributed to its restriction of the antitumor immune response by inhibiting type I IFN signaling (79). The results of this study showed that the RNAss of patients in the high-risk group were notably higher than those in the low-risk group, and the RNAss were distinctly positively correlated with the risk score, indicating that the high-risk score might lead to enhanced tumor stem cell characteristics (26), which may explain the poorer prognosis in the high-risk group. Interestingly, TMB was also elevated in the high-risk group and positively correlated with tumor stemness, whereas high TMB was associated with better treatment response and better prognosis. One possible reason for our analysis is that as TMB increases, so does the neoantigens, one or more of which are more likely to be immunogenic and trigger a T cell response that enhances the antitumor response(80). In summary, we can take a more comprehensive view of the immunotherapeutic effect of PAAD.

Finally, we screened several potential small-molecule drugs for patients with PAAD, with tamoxifen in the top spot. As a selective estrogen receptor modulator (SERM), tamoxifen is clinically used for hormone receptor-positive breast cancer. However, in recent years, tamoxifen may inhibit peritoneal mesothelium-mesenchymal transition, as well as peritoneal mesenchymal cell migration, interstitial fibrosis and neovascularization due to its ability to quiescent the peritoneal stroma (81). This may aid in the immunotherapy of PAADs with desmoplastic stroma. Another SERM, bazedoxifene, was shown to have the function of inhibiting STAT3 phosphorylation and STAT3 DNA binding, inducing apoptosis, and suppressing tumor growth in PAAD cells with persistent IL6/GP130/STAT3 signaling (82). In addition, a non-selective adenosine receptor antagonist caffeine, and its analog CGS 15943 has been reported to block the proliferation in HCC and PDAC cell lines by inhibiting the PI3K/Akt pathway (83). However, the exact function of above small molecule drugs remains unclear and needs to be further confirmed by future prospective studies.

So far, pyroptosis-related prognostic models of PAAD have mushroomed. Similar to these studies, our study also carried out methods such as survival analysis, ROC and external cohort validation of PMSRPS, but it still has its advantages. First, after knowing the importance of subtyping based on molecular characteristics of cancer for improving clinical treatment decision-making, we did not directly use PRGs to establish a prognostic signature as many previous studies had done (84–88), but instead identified three stable pyroptosis molecular subtypes based on PRGs expression levels. By analysis, we found that different PRGclusters had different survival outcomes and immune cell infiltration manifestations. To highlight this difference, we extracted the DEGs between PRGclusters to establish a PMSRPS, which made the modeling process more rigorous. Secondly, there are only three signature genes in this study, which is significantly less than other studies (mostly between 5 and 8), which helps simplify the calculation of risk scores and the risk grouping process for patients with PAAD, thus reducing cost estimates and enabling faster prognosis assessment. Finally, we screened new potential small-molecule drugs for patients with PAAD based on DEGs in the high- and low-risk groups, which provides a reference for PAAD to develop new treatment options. For all this, there are still some shortcomings in this study. One is that despite the combination of the TCGA and GSE102238 cohorts in this study, the PAAD sample size used to construct the PMSRPS is still small, mainly due to the limited number of patients with PAAD with complete clinical information we obtained from current public databases. Another shortcoming is that the transcriptional data for the PRGs included in the merged cohort (TCGA+GSE102238) were incomplete. This situation prevents us from comparing the prediction performance of PMSRPS with other existing PRG models in the TCGA+GSE102238 cohort. In short, a large prospective multicenter study is needed in the future to further verify the exact effect of PMSRPS on the survival and treatment of patients with PAAD.

## 5 Conclusion

In conclusion, we identified a novel signature, PMSRPS for patients with PAAD. This signature is a good predictor of prognosis, immune microenvironment, immunotherapy effect, genomic instability and tumor stemness in patients with PAAD.

## Data availability statement

Publicly available datasets were analyzed in this study. This data can be found here: The TCGA database can be found here: https://portal.gdc.cancer.gov/, the GEO database https://www.ncbi.nlm.nih.gov/geo/, the ICGC database https://dcc.icgc.org/, the GTEx database https://xenabrowser.net/datapages/, and the original data for experimental validation are available through corresponding authors.

## Ethics statement

The studies involving human participants were reviewed and approved by The Second Affiliated Hospital of Nanchang University Medical Research Ethics Committee. The patients/participants provided their written informed consent to participate in this study.

## Author contributions

QH conducted the formal analysis and wrote the original manuscript. XP and JZ collected the samples and conducted the experiments. JX took the raw data from the public database and did the collation. QH and JZ completed the drawing. QL participated in the major revision of the manuscript. HJ guided design, reviewed the manuscript and provided funding acquisition. All authors contributed to the article and approved the submitted version.
