# Supplementary material for: Construction and comprehensive analysis of a novel prognostic signature associated with pyroptosis molecular subtypes in patients with pancreatic adenocarcinoma
Source: Front Immunol. 2023 Feb 3;14:1111494. doi: 10.3389/fimmu.2023.1111494 (PMC9935619; doi:10.3389/fimmu.2023.1111494)
Supplement: Supplementary file 1 [file DataSheet_1.docx]

Supplementary Material

# Supplementary Figures and Tables

## Supplementary Tables

### Supplementary Table 1. Pyroptosis-related genes

| Gene | Full-names |
| --- | --- |
| AIM2 | absent in melanoma 2 |
| BAK1 | BCL2 Antagonist/Killer 1 |
| BAX | BCL2 Associated X |
| CASP1 | cysteine-aspartic acid protease-1 |
| CASP3 | cysteine-aspartic acid protease-3 |
| CASP4 | cysteine-aspartic acid protease-4 |
| CASP5 | cysteine-aspartic acid protease-5 |
| CASP6 | cysteine-aspartic acid protease-6 |
| CASP8 | cysteine-aspartic acid protease-8 |
| CASP9 | cysteine-aspartic acid protease-9 |
| CHMP2A | charged multivesicular body protein 2A |
| CHMP2B | charged multivesicular body protein 2B |
| CHMP3 | charged multivesicular body protein 3 |
| CHMP4A | charged multivesicular body protein 4A |
| CHMP4B | charged multivesicular body protein 4B |
| CHMP4C | charged multivesicular body protein 4C |
| CHMP6 | charged multivesicular body protein 6 |
| CHMP7 | charged multivesicular body protein 7 |
| CYCS | cytochrome c, somatic |
| ELANE | elastase, neutrophil expressed |
| GPX4 | glutathione peroxidase 4 |
| GSDMA | gasdermin A |
| GSDMB | gasdermin B |
| GSDMC | gasdermin C |
| GSDMD | gasdermin D |
| GSDME | gasdermin E |
| GZMA | granzyme A |
| GZMB | granzyme B |
| HMGB1 | high mobility group box 1 |
| IL18 | interleukin 18 |
| IL1A | interleukin 1, alpha |
| IL1B | interleukin 1 beta |
| IL6 | interleukin 6 |
| IRF1 | interferon regulatory factor 1 |
| IRF2 | interferon regulatory factor 2 |
| NLRC4 | NLR family CARD domain containing 4 |
| NLRP1 | NLR family pyrin domain containing 1 |
| NLRP2 | NLR family pyrin domain containing 2 |
| NLRP3 | NLR family pyrin domain containing 3 |
| NLRP6 | NLR family pyrin domain containing 6 |
| NLRP7 | NLR family pyrin domain containing 7 |
| NOD1 | nucleotide binding oligomerization domain containing 1 |
| NOD2 | nucleotide binding oligomerization domain containing 2 |
| PJVK | pejvakin/deafness, autosomal recessive 59 |
| PLCG1 | phospholipase C gamma 1 |
| PRKACA | protein kinase cAMP-activated catalytic subunit alpha |
| PYCARD | PYD and CARD domain containing |
| SCAF11 | SR-related CTD associated factor 11 |
| TIRAP | TIR domain containing adaptor protein |
| TNF | tumor necrosis factor |
| TP53 | tumor protein p53 |
| TP63 | tumor protein p63 |

### Supplementary Table 2. Immune checkpoint genes

| Gene | Full-names |
| --- | --- |
| ADORA2A | Adenosine A2a Receptor |
| BTLA | B And T Lymphocyte Associated |
| BTNL2 | Butyrophilin Like 2 |
| CD200 | CD200 Molecule |
| CD200R1 | CD200 Receptor 1 |
| CD244 | CD244 Molecule |
| CD27 | CD27 Molecule |
| CD274 | CD274 Molecule |
| CD276 | CD276 Molecule |
| CD28 | CD28 Molecule |
| CD40 | CD40 Molecule |
| CD40LG | CD40 Ligand |
| CD44 | CD44 Molecule |
| CD48 | CD48 Molecule |
| CD70 | CD70 Molecule |
| CD80 | CD80 Molecule |
| CD86 | CD86 Molecule |
| CTLA4 | Cytotoxic T-Lymphocyte Associated Protein 4 |
| HAVCR2 | Hepatitis A Virus Cellular Receptor 2 |
| HHLA2 | HERV-H LTR-Associating 2 |
| ICOS | Inducible T Cell Costimulator |
| ICOSLG | Inducible T Cell Costimulator Ligand |
| IDO1 | Indoleamine 2,3-Dioxygenase 1 |
| LAG3 | Lymphocyte Activating 3 |
| LAIR1 | Leukocyte Associated Immunoglobulin Like Receptor 1 |
| LGALS9 | Galectin 9 |
| NRP1 | Neuropilin 1 |
| PDCD1 | Programmed Cell Death 1 |
| PDCD1LG2 | Programmed Cell Death 1 Ligand 2 |
| TIGIT | T Cell Immunoreceptor With Ig And ITIM Domains |
| TNFRSF4 | TNF Receptor Superfamily Member 4 |
| TNFRSF9 | TNF Receptor Superfamily Member 9 |
| TNFRSF14 | TNF Receptor Superfamily Member 14 |
| TNFRSF18 | TNF Receptor Superfamily Member 18 |
| TNFRSF25 | TNF Receptor Superfamily Member 25 |
| TNFSF4 | TNF Superfamily Member 4 |
| TNFSF9 | TNF Superfamily Member 9 |
| TNFSF14 | TNF Superfamily Member 14 |
| TNFSF15 | TNF Superfamily Member 15 |
| VTCN1 | V-Set Domain Containing T Cell Activation Inhibitor 1 |

### Supplementary Table 3. Primers and siRNA target sequences

| **Name** | **Sequences (5'-3')** |
| --- | --- |
| **Primers for real-time PCR** | |
| SFTA2 sense: | AACAGGGCCGGGTATGACTT |
| SFTA2 antisense: | AGGAGGAGGCAGAGCTTTTCAA |
| GAPDH sense: | AGAAGGCTGGGGCTCATTTG |
| GAPDH antisense: | AGGGGCCATCCACAGTCTTC |
| **The target sites of siRNA** | |
| si-SFTA2#1 sense: | GCCGGGUAUGACUUUGCAATT |
| si-SFTA2#1 antisense: | UUGCAAAGUCAUACCCGGCTT |
| si-SFTA2#2 sense: | CCACCAUGCAAGAUCUCAATT |
| si-SFTA2#2 antisense: | UUGAGAUCUUGCAUGGUGGTT |
| si-SFTA2#3 sense: | CUGAGCGGCAAUAAAUAAATT |
| si-SFTA2#3 antisense: | UUUAUUUAUUGCCGCUCAGTT |

### Supplementary Table 4. Differentially expressed genes associated with prognosis

| Gene | HR | HR.95L | HR.95H | *P* value |
| --- | --- | --- | --- | --- |
| FCGR2B | 1.248843169 | 1.032035599 | 1.511197155 | 0.022366841 |
| GBP1P1 | 1.34546795 | 1.07081721 | 1.690563045 | 0.010855401 |
| EFEMP1 | 1.15845001 | 1.011980429 | 1.326118952 | 0.032954018 |
| CLEC2B | 1.381935561 | 1.129727488 | 1.690448286 | 0.001653153 |
| HLA-DRA | 1.183676572 | 1.011306327 | 1.385426146 | 0.035730828 |
| C1S | 1.185385547 | 1.013846833 | 1.385947905 | 0.032974731 |
| CHSY1 | 1.300564665 | 1.038141071 | 1.629324274 | 0.022283598 |
| GFPT2 | 1.203726913 | 1.052382241 | 1.376836689 | 0.006836393 |
| HSD11B1 | 1.203668032 | 1.048661956 | 1.381586051 | 0.008401467 |
| GBP1 | 1.301804336 | 1.096725244 | 1.545231624 | 0.002564661 |
| CCDC80 | 1.175055828 | 1.037609977 | 1.330708291 | 0.011032406 |
| HAVCR2 | 1.221985303 | 1.037033909 | 1.439922137 | 0.016653916 |
| FCER1G | 1.225217391 | 1.040460561 | 1.44278189 | 0.014868635 |
| STK17B | 1.265822197 | 1.033349315 | 1.550594568 | 0.022796433 |
| EPS8L1 | 1.175883935 | 1.042605922 | 1.326199094 | 0.008296725 |
| TNFSF13B | 1.222514765 | 1.016832328 | 1.469802159 | 0.032552545 |
| ICAM1 | 1.186679527 | 1.012105059 | 1.391365736 | 0.035016564 |
| ATP11C | 1.409230312 | 1.085480886 | 1.829539422 | 0.009999025 |
| PKP3 | 1.181571246 | 1.009188265 | 1.383399566 | 0.038112836 |
| APOL3 | 1.296174832 | 1.022442307 | 1.643192171 | 0.032086235 |
| PMP22 | 1.287130086 | 1.064384594 | 1.556489888 | 0.009226043 |
| F13A1 | 1.132840402 | 1.004800912 | 1.277195672 | 0.041526957 |
| HLA-DMB | 1.177901463 | 1.012063891 | 1.370913306 | 0.034442465 |
| CLMP | 1.170724314 | 1.020703242 | 1.342795206 | 0.0242688 |
| MSR1 | 1.198715195 | 1.034427483 | 1.389095072 | 0.015951099 |
| BAIAP2L1 | 1.221867742 | 1.005035093 | 1.485481242 | 0.044393877 |
| CD300A | 1.207812531 | 1.012446644 | 1.440877025 | 0.035960665 |
| C1R | 1.213097227 | 1.023282122 | 1.438122342 | 0.026077262 |
| C1orf116 | 1.244511467 | 1.021009905 | 1.516938067 | 0.030323911 |
| THBS1 | 1.18057238 | 1.041096266 | 1.338734169 | 0.009658807 |
| FA2H | 1.210762876 | 1.06524048 | 1.376165072 | 0.003418895 |
| BCL2A1 | 1.185485907 | 1.028053957 | 1.367026337 | 0.019255442 |
| CGN | 1.206595284 | 1.024479267 | 1.421085059 | 0.024470096 |
| LPCAT4 | 1.267126498 | 1.070792703 | 1.499458819 | 0.005846484 |
| RNF39 | 1.162875842 | 1.014634408 | 1.332775839 | 0.030099787 |
| FUT3 | 1.204005358 | 1.063413806 | 1.363184203 | 0.003384516 |
| VSIG2 | 1.117325419 | 1.021658499 | 1.22195048 | 0.015134446 |
| MKRN2OS | 1.38385458 | 1.109388783 | 1.72622396 | 0.003972424 |
| CXCL9 | 1.137854739 | 1.034941387 | 1.251001673 | 0.007584356 |
| MST1R | 1.216155217 | 1.058457797 | 1.397347647 | 0.005749491 |
| C6orf132 | 1.377175399 | 1.137192061 | 1.667802779 | 0.001053032 |
| TLR1 | 1.327711227 | 1.06295566 | 1.658410759 | 0.01248938 |
| GSDMB | 1.175489995 | 1.020138684 | 1.354498903 | 0.02537407 |
| RGS10 | 1.340092322 | 1.066472902 | 1.683912856 | 0.011995646 |
| ERN2 | 1.110464704 | 1.003534299 | 1.228788952 | 0.042533841 |
| LDLRAD3 | 1.345168909 | 1.067904465 | 1.694420665 | 0.011808188 |
| SH2D3A | 1.256864404 | 1.060188314 | 1.490025977 | 0.008460473 |
| FAM83H | 1.199962117 | 1.005873161 | 1.431501642 | 0.042865649 |
| DOK4 | 1.309490358 | 1.031202815 | 1.662878508 | 0.026964582 |
| CD74 | 1.231189344 | 1.00263786 | 1.511839182 | 0.047132417 |
| ST14 | 1.296399662 | 1.056708664 | 1.590459264 | 0.012817717 |
| OCIAD2 | 1.347859067 | 1.074604092 | 1.690598498 | 0.009811376 |
| BIK | 1.249600794 | 1.074011354 | 1.453897242 | 0.0039248 |
| SH2D4A | 1.373547499 | 1.133589148 | 1.664300277 | 0.001195723 |
| REPS2 | 1.383525687 | 1.109932328 | 1.724558586 | 0.003880211 |
| TSPAN15 | 1.284323357 | 1.0606831 | 1.555117155 | 0.010362303 |
| MUC5AC | 1.088714808 | 1.019335806 | 1.16281595 | 0.011405872 |
| JUP | 1.243130876 | 1.016193014 | 1.520748869 | 0.034331581 |
| IL1R1 | 1.205160922 | 1.016710608 | 1.428541058 | 0.031477281 |
| SDCBP2 | 1.246877856 | 1.081095038 | 1.438082993 | 0.002436079 |
| LAD1 | 1.212464285 | 1.049145375 | 1.401206809 | 0.009057059 |
| TNFAIP6 | 1.156342408 | 1.017790843 | 1.313754956 | 0.025696071 |
| GPR68 | 1.204120725 | 1.0139256 | 1.429993206 | 0.034206698 |
| ADAM12 | 1.139985375 | 1.002846309 | 1.295878186 | 0.04513152 |
| DCN | 1.158823912 | 1.003093841 | 1.338731038 | 0.045293703 |
| FUT2 | 1.160953691 | 1.010172304 | 1.334241166 | 0.035505167 |
| SLC1A3 | 1.202613153 | 1.030609585 | 1.403323254 | 0.019138156 |
| KRT8 | 1.373133772 | 1.139737873 | 1.654324562 | 0.000849797 |
| MILR1 | 1.248492163 | 1.046104806 | 1.490034911 | 0.013914579 |
| FSTL1 | 1.228901069 | 1.03354428 | 1.461183489 | 0.019622524 |
| MUC1 | 1.181847051 | 1.056980506 | 1.321464723 | 0.003360673 |
| CD80 | 1.373305496 | 1.080497414 | 1.745462748 | 0.009520983 |
| APOL4 | 1.261092819 | 1.038878172 | 1.530838881 | 0.0189951 |
| B3GNT3 | 1.278287228 | 1.086318159 | 1.504180173 | 0.003104691 |
| ARL14 | 1.1653562 | 1.053005962 | 1.289693622 | 0.003091354 |
| GPNMB | 1.148236081 | 1.005724972 | 1.310940998 | 0.040914474 |
| MDFIC | 1.319404034 | 1.07478829 | 1.61969294 | 0.008065047 |
| CAPN5 | 1.201294677 | 1.027913816 | 1.403920134 | 0.02110193 |
| TUBB6 | 1.234682499 | 1.020194346 | 1.494265167 | 0.030363323 |
| VILL | 1.199855723 | 1.048829471 | 1.372629007 | 0.007941335 |
| PARD6B | 1.367783814 | 1.120729931 | 1.669298293 | 0.002060504 |
| SERPINE1 | 1.195029155 | 1.051090576 | 1.35867899 | 0.006510242 |
| FCGR3A | 1.175897157 | 1.026251358 | 1.347363988 | 0.019643983 |
| ERBB3 | 1.223545382 | 1.039526155 | 1.440140101 | 0.01526115 |
| CXCL10 | 1.189038856 | 1.068056908 | 1.323724785 | 0.001563768 |
| SGK1 | 1.226364449 | 1.009342296 | 1.490049281 | 0.040019662 |
| HLA-DRB1 | 1.20303028 | 1.019999884 | 1.41890394 | 0.028154206 |
| RHBDL2 | 1.316338632 | 1.103525613 | 1.570192276 | 0.002251739 |
| GALE | 1.20654241 | 1.018014097 | 1.429984704 | 0.030317398 |
| CCN4 | 1.217747554 | 1.052834212 | 1.408492513 | 0.007967976 |
| ABCC3 | 1.228303244 | 1.065596627 | 1.415853636 | 0.00456399 |
| LIPH | 1.311581113 | 1.125838365 | 1.527968018 | 0.000499055 |
| EVPL | 1.152959683 | 1.012216898 | 1.313271921 | 0.032131454 |
| LINC02041 | 1.261744816 | 1.098290776 | 1.449525041 | 0.001021896 |
| AC130456.2 | 1.235027666 | 1.044032817 | 1.460963019 | 0.013790491 |
| PLS1 | 1.297115854 | 1.111205611 | 1.514129809 | 0.000980994 |
| MSN | 1.451870857 | 1.15634532 | 1.822923437 | 0.001322908 |
| TMC5 | 1.207300908 | 1.065625112 | 1.367812626 | 0.003096586 |
| LINC01094 | 1.273678325 | 1.036073697 | 1.565773245 | 0.021654806 |
| FBN1 | 1.172694141 | 1.019017946 | 1.349545956 | 0.026226711 |
| HLA-DMA | 1.249689708 | 1.031652902 | 1.51380795 | 0.022695852 |
| SLC44A4 | 1.141200158 | 1.008637361 | 1.291185366 | 0.036040208 |
| F11R | 1.255952111 | 1.010183926 | 1.561513369 | 0.040251281 |
| SLC45A3 | 1.189269992 | 1.031206093 | 1.371562021 | 0.01720543 |
| PRSS22 | 1.178326641 | 1.0313504 | 1.34624825 | 0.015774366 |
| CHST11 | 1.280397532 | 1.059017474 | 1.548055512 | 0.010710363 |
| IFI16 | 1.458526524 | 1.174321254 | 1.811514195 | 0.000642286 |
| CLIC4 | 1.332473147 | 1.083911012 | 1.638035474 | 0.006432447 |
| TMPRSS4 | 1.170901019 | 1.054574047 | 1.300059678 | 0.003123855 |
| MFAP5 | 1.167507838 | 1.044095635 | 1.305507376 | 0.00658797 |
| RAB31 | 1.238555672 | 1.047953071 | 1.463825238 | 0.012096165 |
| PLA2G7 | 1.166939722 | 1.012632685 | 1.34476038 | 0.032888521 |
| CD248 | 1.181992829 | 1.005317524 | 1.389717193 | 0.04295286 |
| LMO7 | 1.324538944 | 1.092541479 | 1.605800282 | 0.004225268 |
| CILP2 | 1.14231693 | 1.003930608 | 1.299779045 | 0.043434855 |
| TRPM4 | 1.245111474 | 1.035013506 | 1.497857346 | 0.020075255 |
| KCNK1 | 1.226781038 | 1.045400513 | 1.439631697 | 0.012284086 |
| RNF223 | 1.366992297 | 1.139157644 | 1.640394506 | 0.000777849 |
| MMEL1 | 1.289274039 | 1.070530278 | 1.552714186 | 0.007396976 |
| NGEF | 1.21776256 | 1.031281129 | 1.437964501 | 0.02016976 |
| CRYBG2 | 1.208933826 | 1.036772943 | 1.409682808 | 0.015490904 |
| ANXA10 | 1.092188767 | 1.007128855 | 1.184432653 | 0.033033193 |
| KPNA7 | 1.233798667 | 1.043120406 | 1.459332157 | 0.014173701 |
| RASAL1 | 1.211392944 | 1.027171269 | 1.42865451 | 0.022697652 |
| PLXDC2 | 1.199509321 | 1.011277173 | 1.4227777 | 0.03673257 |
| F2RL1 | 1.269078314 | 1.059760987 | 1.519738684 | 0.009566617 |
| BCL2L15 | 1.203265266 | 1.051055353 | 1.377517651 | 0.007327283 |
| SCNN1A | 1.184776578 | 1.059807454 | 1.324481664 | 0.002869966 |
| STX19 | 1.20249586 | 1.012393292 | 1.428295016 | 0.035706651 |
| CYP2S1 | 1.099689113 | 1.001413352 | 1.207609367 | 0.046642337 |
| COL15A1 | 1.184587224 | 1.002256037 | 1.400088239 | 0.0469901 |
| MICB | 1.30464661 | 1.078917858 | 1.577601821 | 0.006076385 |
| GBP4 | 1.251781126 | 1.052909724 | 1.488214947 | 0.010957875 |
| ITGAM | 1.202623445 | 1.014220039 | 1.426025018 | 0.033805284 |
| PLPP2 | 1.240496061 | 1.055198354 | 1.458332902 | 0.009031029 |
| VSIG1 | 1.086048049 | 1.003237783 | 1.175693724 | 0.041365653 |
| P2RY6 | 1.29652748 | 1.057124044 | 1.590147832 | 0.01265511 |
| FOXQ1 | 1.176129176 | 1.038613048 | 1.331852937 | 0.010553362 |
| CLDN4 | 1.184584131 | 1.028795404 | 1.363963677 | 0.018544168 |
| TMEM92 | 1.26739483 | 1.085433826 | 1.479859589 | 0.002729184 |
| SOD2 | 1.292967509 | 1.070357937 | 1.561874698 | 0.007692811 |
| HLA-DRB5 | 1.161384449 | 1.021496379 | 1.320429388 | 0.022326909 |
| SLC6A20 | 1.191432055 | 1.059868572 | 1.33932676 | 0.003347262 |
| ST6GALNAC1 | 1.110030247 | 1.008859258 | 1.221346921 | 0.032285609 |
| TMC7 | 1.280996494 | 1.073514981 | 1.528578592 | 0.006017714 |
| STYK1 | 1.215679665 | 1.043564892 | 1.416181264 | 0.012160931 |
| PPP1R13L | 1.194418514 | 1.005310827 | 1.419098997 | 0.043363221 |
| KRT19 | 1.277172476 | 1.120592884 | 1.455630816 | 0.000246199 |
| PLEK2 | 1.301439456 | 1.13043425 | 1.498313288 | 0.000246596 |
| TSPAN1 | 1.201538692 | 1.055968188 | 1.367176819 | 0.005328964 |
| PIM1 | 1.253836693 | 1.044249769 | 1.505488915 | 0.01535354 |
| FUT6 | 1.167286671 | 1.007827133 | 1.351976075 | 0.039017839 |
| KCP | 1.217205802 | 1.039003148 | 1.425972546 | 0.014945105 |
| MYC | 1.237579903 | 1.009023191 | 1.517907646 | 0.040737384 |
| FAP | 1.21820321 | 1.041258535 | 1.425216707 | 0.013706751 |
| POF1B | 1.195308864 | 1.046082064 | 1.365823322 | 0.008738546 |
| HKDC1 | 1.246264846 | 1.078373014 | 1.440295747 | 0.002863724 |
| CTSK | 1.153850092 | 1.004629849 | 1.325234399 | 0.042832618 |
| GPRC5A | 1.25969726 | 1.124123873 | 1.411621285 | 7.07E-05 |
| MROH6 | 1.245221145 | 1.076499888 | 1.44038631 | 0.003154425 |
| NECTIN4 | 1.16952467 | 1.031264697 | 1.326320931 | 0.014704971 |
| MN1 | 1.229513505 | 1.01594456 | 1.4879783 | 0.033799112 |
| SEMA4B | 1.253141953 | 1.027996667 | 1.527597125 | 0.025533708 |
| HLA-DQB1 | 1.147644417 | 1.00191789 | 1.314566513 | 0.046853867 |
| KLF5 | 1.208006376 | 1.057944754 | 1.379353128 | 0.005233809 |
| LINC01133 | 1.187604338 | 1.075432449 | 1.311476202 | 0.000682337 |
| SLC37A2 | 1.219481778 | 1.022071263 | 1.455021642 | 0.027646872 |
| C3 | 1.154544153 | 1.021987694 | 1.304293789 | 0.020915818 |
| SDR16C5 | 1.214040762 | 1.085612912 | 1.357661607 | 0.000674059 |
| MMP2 | 1.180434633 | 1.046178468 | 1.331919902 | 0.007085676 |
| AL355312.3 | 1.162177749 | 1.028035756 | 1.313823096 | 0.016313306 |
| PERP | 1.33049295 | 1.09400504 | 1.618101768 | 0.004239637 |
| NMU | 1.132357564 | 1.028098241 | 1.247189812 | 0.011660605 |
| CTSE | 1.133215881 | 1.042246443 | 1.232125321 | 0.003399205 |
| PHLDA2 | 1.159922481 | 1.032384617 | 1.303216011 | 0.012551724 |
| CLIC3 | 1.135519854 | 1.025455621 | 1.257397505 | 0.014557654 |
| GCKR | 1.21869587 | 1.058630287 | 1.402963473 | 0.005904305 |
| ALDH3B1 | 1.338857822 | 1.133164534 | 1.581888785 | 0.000606022 |
| CYBRD1 | 1.182219919 | 1.008931055 | 1.385271996 | 0.038460704 |
| IQGAP3 | 1.222669723 | 1.049659938 | 1.424195778 | 0.009806604 |
| MSLN | 1.144850587 | 1.06169743 | 1.23451638 | 0.000437928 |
| CD300LF | 1.18732365 | 1.005408624 | 1.402153728 | 0.043018145 |
| PDPN | 1.195232814 | 1.036765469 | 1.377921549 | 0.013991112 |
| LUM | 1.204731037 | 1.045057844 | 1.388800513 | 0.010243887 |
| NNMT | 1.234913278 | 1.036686494 | 1.47104338 | 0.018100582 |
| RHPN2 | 1.243774776 | 1.05423954 | 1.467385387 | 0.00970605 |
| GALNT6 | 1.175738759 | 1.017233631 | 1.358942122 | 0.02843518 |
| SFTA2 | 1.214071538 | 1.104525645 | 1.334482098 | 5.81E-05 |
| LTBP2 | 1.226144732 | 1.035434967 | 1.451980039 | 0.018094563 |
| SPDEF | 1.136825357 | 1.024348304 | 1.261652787 | 0.015842064 |
| RARRES1 | 1.168702877 | 1.033163611 | 1.322023346 | 0.013185947 |
| COL6A3 | 1.229430506 | 1.071923953 | 1.410080786 | 0.003147888 |
| SRPX2 | 1.258582534 | 1.071723032 | 1.478021792 | 0.00503659 |
| RAB27B | 1.289191431 | 1.093664032 | 1.5196756 | 0.002471198 |
| S100A14 | 1.133376544 | 1.033401836 | 1.243023136 | 0.007876629 |
| TNFSF4 | 1.220351182 | 1.021795301 | 1.457490561 | 0.027953402 |
| PXDN | 1.180676226 | 1.000728593 | 1.392981435 | 0.048999423 |
| HS3ST1 | 1.248308369 | 1.081726363 | 1.440543409 | 0.00240576 |
| TSPAN8 | 1.168302012 | 1.053426987 | 1.29570403 | 0.00322355 |
| SPARC | 1.174583488 | 1.004457418 | 1.373524001 | 0.043833918 |
| MRC2 | 1.182606704 | 1.014329522 | 1.378801057 | 0.032222472 |
| VCAN | 1.173764618 | 1.031087522 | 1.336184707 | 0.015395519 |
| S100P | 1.12228747 | 1.041127835 | 1.209773789 | 0.002592472 |
| ANOS1 | 1.19199653 | 1.000215312 | 1.420549865 | 0.049719483 |
| MMP28 | 1.294728921 | 1.117702598 | 1.499793399 | 0.000574565 |
| NQO1 | 1.161313012 | 1.01748815 | 1.325467931 | 0.026625134 |
| XDH | 1.191876837 | 1.048442104 | 1.354934515 | 0.007295427 |
| SEMA3C | 1.285877659 | 1.114237355 | 1.483957926 | 0.00058227 |
| NCAM1 | 0.715079542 | 0.594474603 | 0.860152392 | 0.000373092 |
| TFCP2L1 | 1.280027654 | 1.0999801 | 1.489545851 | 0.001412745 |
| AP005233.2 | 1.136577868 | 1.029318882 | 1.255013654 | 0.011362676 |
| DUSP5 | 1.178561978 | 1.011185733 | 1.373643131 | 0.035526937 |
| COL5A2 | 1.173674282 | 1.026616276 | 1.341797664 | 0.019050111 |
| BNIP5 | 1.149500212 | 1.021761783 | 1.293208221 | 0.020440741 |
| FOXA2 | 0.87192966 | 0.760882938 | 0.999183045 | 0.048641291 |
| ARL4C | 1.244293059 | 1.048678431 | 1.476396548 | 0.012257835 |
| LEMD1 | 1.155812134 | 1.038698717 | 1.286130105 | 0.007894951 |
| AKR1B10 | 1.080238361 | 1.002177787 | 1.164379147 | 0.043715437 |
| ERO1B | 0.874724523 | 0.769450144 | 0.994402297 | 0.040779797 |
| PTGIS | 1.147454365 | 1.026666302 | 1.282453234 | 0.015363514 |
| SLC16A5 | 1.205441738 | 1.047819859 | 1.386774426 | 0.008967563 |
| CDH11 | 1.17595379 | 1.020788249 | 1.354705364 | 0.024771473 |
| PROM2 | 1.142346574 | 1.027631772 | 1.26986702 | 0.013710079 |
| FAM83B | 1.278266441 | 1.083630042 | 1.50786249 | 0.003580635 |
| ANXA3 | 1.414610274 | 1.181309715 | 1.693986091 | 0.00016197 |
| METTL7B | 1.150105211 | 1.00416033 | 1.317261752 | 0.043390885 |
| CCL18 | 1.10883701 | 1.015907854 | 1.210266767 | 0.020702227 |
| COL17A1 | 1.192248596 | 1.098619778 | 1.29385684 | 2.51E-05 |
| LCN2 | 1.123076796 | 1.035765308 | 1.217748344 | 0.00493903 |
| SSTR2 | 0.773992857 | 0.640456179 | 0.935372259 | 0.008015753 |
| DUOX2 | 1.112043163 | 1.029280807 | 1.201460271 | 0.007116148 |
| GREM1 | 1.154408472 | 1.037242352 | 1.284809589 | 0.008548262 |
| MYEOV | 1.209524171 | 1.087041678 | 1.345807387 | 0.000479293 |
| PLAC8 | 1.204149145 | 1.055970068 | 1.373121461 | 0.005557225 |
| HHLA2 | 1.18243928 | 1.054542995 | 1.325846984 | 0.004114352 |
| SFN | 1.156453107 | 1.045925187 | 1.278661041 | 0.004567806 |
| SLCO4A1 | 1.176659044 | 1.027058736 | 1.348049977 | 0.019037543 |
| SCUBE2 | 1.170265045 | 1.001573828 | 1.367368274 | 0.047729912 |
| COL3A1 | 1.152058449 | 1.017767947 | 1.304068058 | 0.025189794 |
| TRIM54 | 1.129465977 | 1.003820564 | 1.270838076 | 0.043038226 |
| PROM1 | 1.237356681 | 1.089477595 | 1.405307977 | 0.001039441 |
| ATF3 | 1.176267128 | 1.006721974 | 1.374365903 | 0.040921293 |
| ADGRF1 | 1.232385724 | 1.083673554 | 1.401505616 | 0.001448999 |
| TACSTD2 | 1.150442324 | 1.031406236 | 1.28321654 | 0.011907763 |
| PTPRR | 1.315581772 | 1.135708625 | 1.523943166 | 0.000255748 |
| FOXL1 | 1.164689953 | 1.032034677 | 1.314396422 | 0.013471625 |
| FERMT1 | 1.247868549 | 1.076660994 | 1.44630104 | 0.00327168 |
| LAMB3 | 1.220676881 | 1.087114431 | 1.37064876 | 0.000744263 |
| GCNT3 | 1.138333614 | 1.018577233 | 1.272170018 | 0.022341295 |
| SFRP2 | 1.153767405 | 1.038657472 | 1.281634475 | 0.00764723 |
| COL8A1 | 1.16830134 | 1.032255982 | 1.322276689 | 0.01379526 |
| PIGR | 1.089981785 | 1.010666054 | 1.175522111 | 0.0254043 |
| KLK10 | 1.201333343 | 1.08983384 | 1.324240216 | 0.000223449 |
| KCNN4 | 1.191974642 | 1.061338627 | 1.338690133 | 0.003025673 |
| TRIM29 | 1.146223245 | 1.051799185 | 1.249124117 | 0.001862515 |
| CELF3 | 0.803870062 | 0.704773084 | 0.916900901 | 0.001144249 |
| SPRR1A | 1.089670492 | 1.010089749 | 1.175521067 | 0.026457897 |
| PADI1 | 1.118262251 | 1.037483911 | 1.205329981 | 0.003479009 |
| TCN1 | 1.138181801 | 1.050076484 | 1.233679483 | 0.001640377 |
| ITGB6 | 1.270170093 | 1.12626651 | 1.432460301 | 9.69E-05 |
| SERPINB5 | 1.236915133 | 1.116886298 | 1.369843151 | 4.45E-05 |
| SNAP25 | 0.809926838 | 0.708729435 | 0.925573922 | 0.001963522 |
| CXCL8 | 1.136380227 | 1.02595875 | 1.258686103 | 0.014232394 |
| PPP1R1A | 0.868452189 | 0.77650046 | 0.971292669 | 0.013508635 |
| KRT7 | 1.309509283 | 1.15565036 | 1.483852402 | 2.35E-05 |
| AC136475.3 | 1.140505124 | 1.019589132 | 1.275760891 | 0.021491517 |
| COL12A1 | 1.172944544 | 1.041348471 | 1.321170524 | 0.008607113 |
| MUC5B | 1.083204041 | 1.000977146 | 1.172185598 | 0.047232304 |
| MUC4 | 1.11633716 | 1.014602774 | 1.228272471 | 0.02398817 |
| SPRR3 | 1.126702368 | 1.040707004 | 1.219803673 | 0.003230022 |
| CST1 | 1.085044203 | 1.004924835 | 1.171551226 | 0.03702489 |
| KRT16 | 1.128374374 | 1.044328609 | 1.219183997 | 0.002226302 |
| TNNT1 | 1.124385326 | 1.025845957 | 1.23239006 | 0.012236287 |
| PSCA | 1.139043692 | 1.062899989 | 1.220642153 | 0.000226019 |
| SPRR1B | 1.136891103 | 1.062979577 | 1.215941874 | 0.000183479 |
| AQP5 | 1.132654176 | 1.038710866 | 1.235093927 | 0.004806613 |

## Supplementary Figures


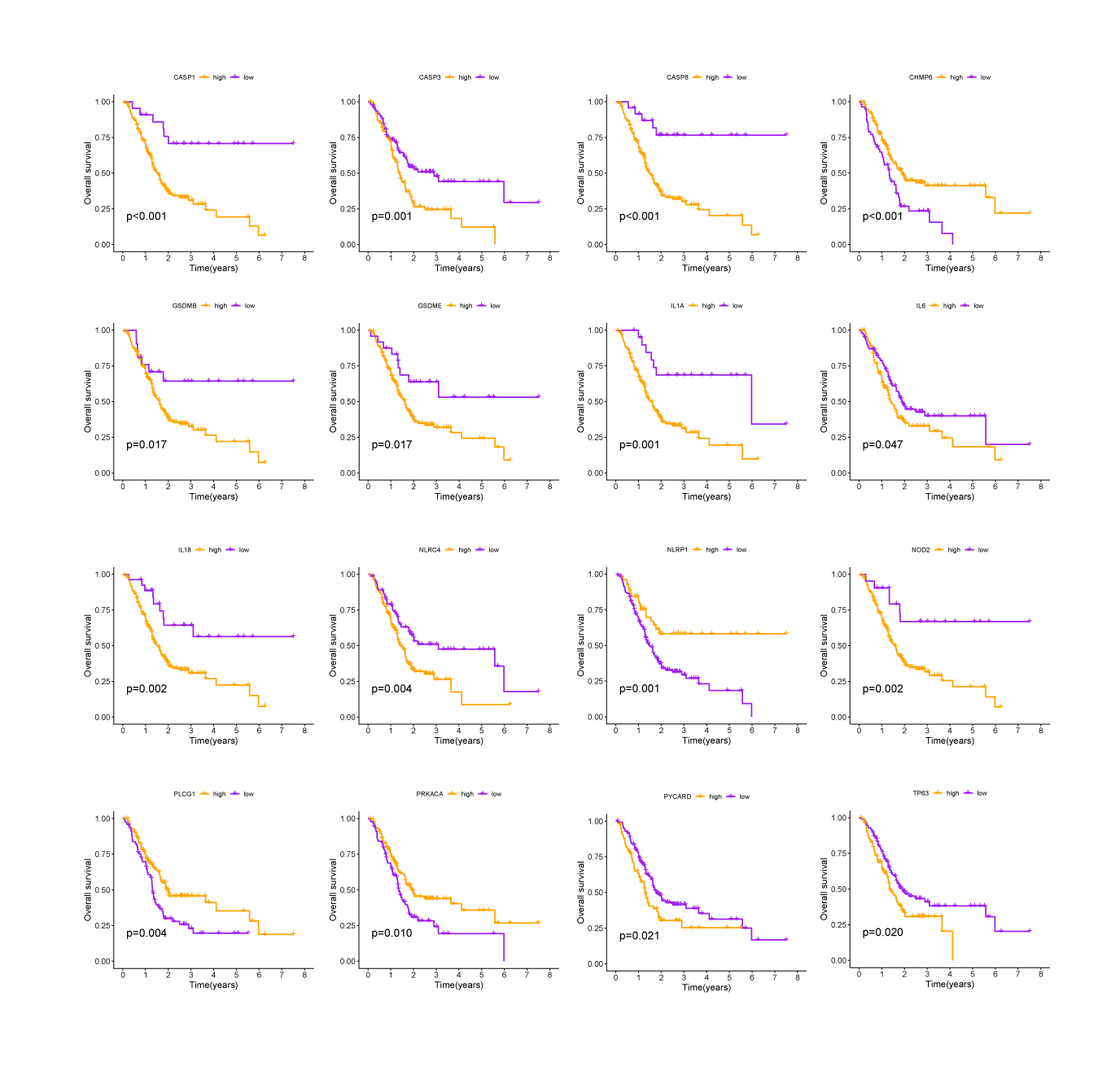


**Supplementary Figure 1.** **Results of Kaplan-Meier survival analysis of pyroptosis-related genes.**


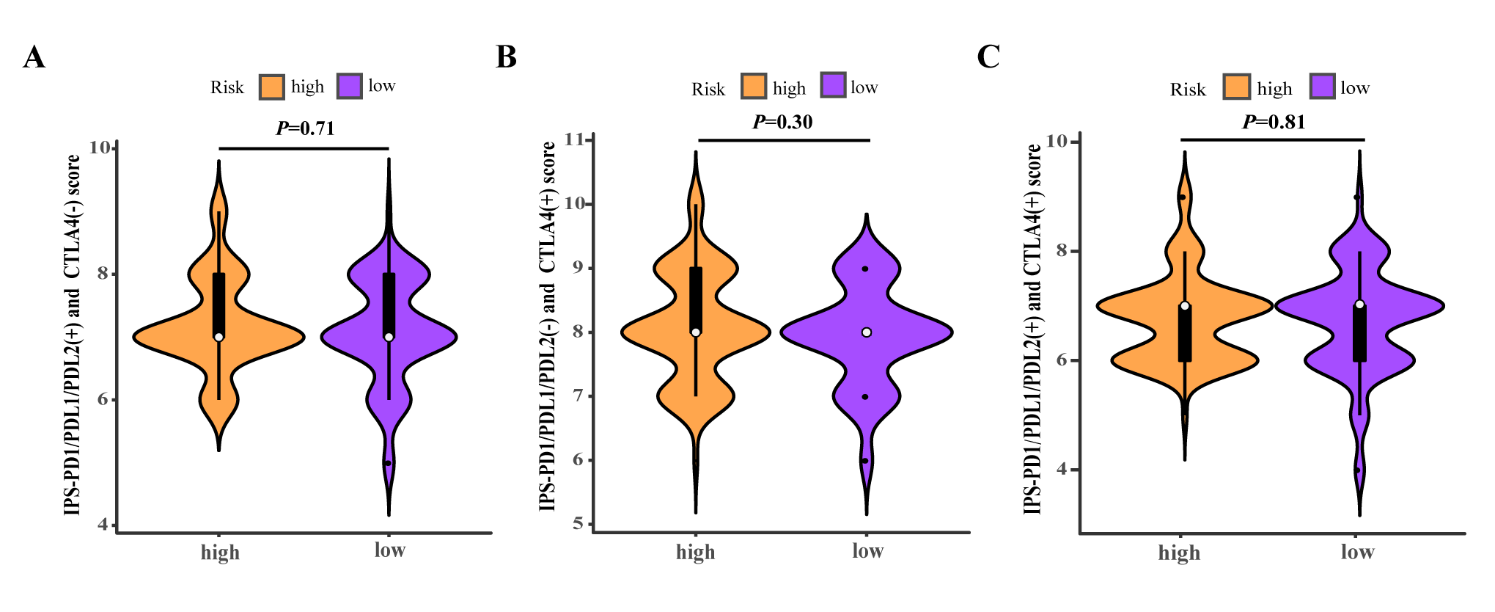


**Supplementary Figure 2.** **Prediction of immunotherapy response by PTRPS. (A)** Comparison of IPS-PD1/PDL1/PDL2(+) and CTLA4(-) scores between the high- and low-risk groups. **(B)** Comparison of IPS-PD1/PDL1/PDL2(-) and CTLA4(+) scores between the high- and low-risk groups. **(C)** Comparison of IPS-PD1/PDL1/PDL2(+) and CTLA4(+) scores between the high- and low-risk groups.

**
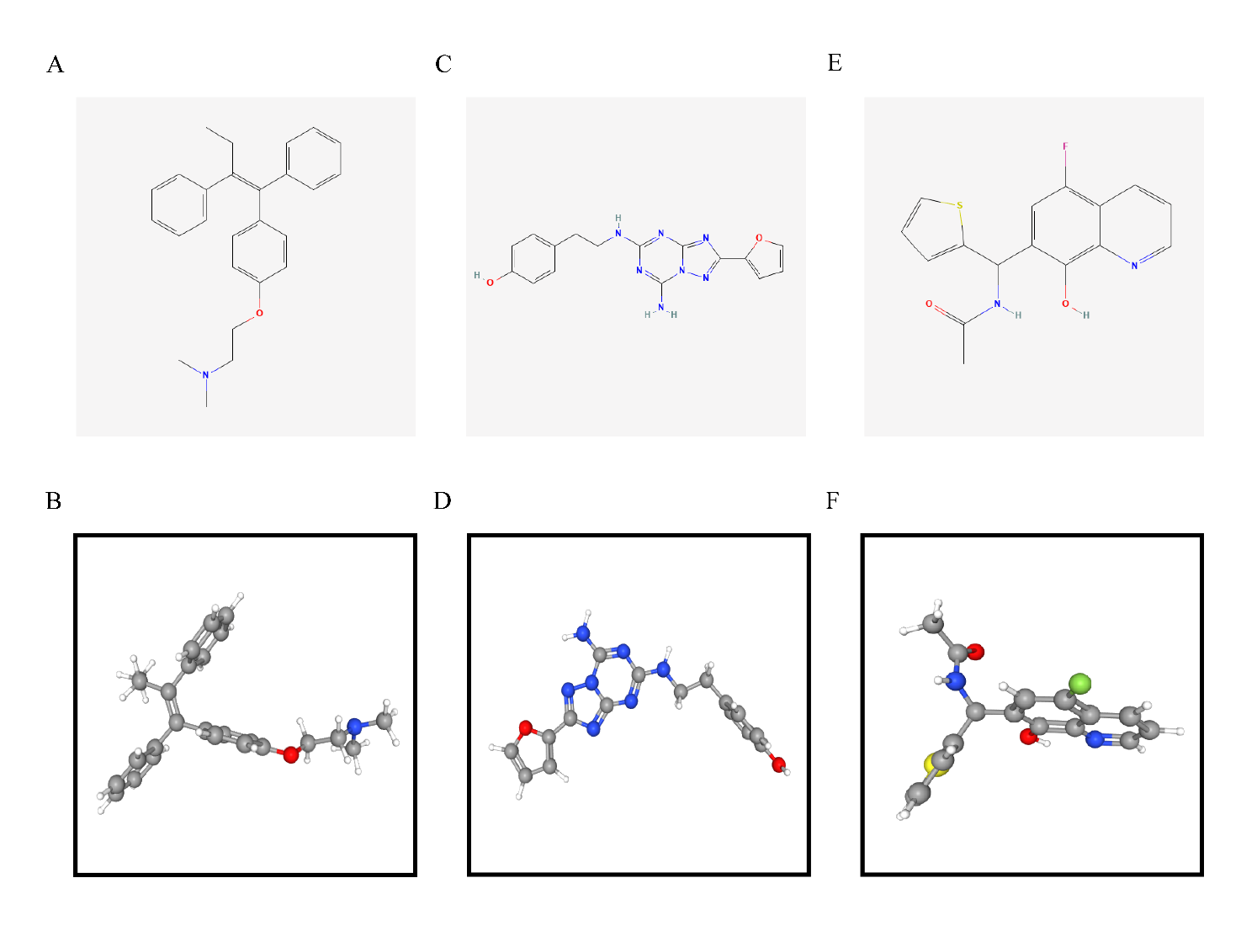
**

**Supplementary Figure 3. Top three potential small molecule drugs screened based on differentially expressed genes between the high- and low-risk groups.** 2D and 3D molecular images of tamoxifen**(A, B)**, ZM-241385**(C, D)** and BRD-A24021119**(E, F)**.
